# Variability in drought stress response in a panel of 100 faba bean genotypes

**DOI:** 10.3389/fpls.2023.1236147

**Published:** 2023-08-30

**Authors:** Christiane Balko, Ana M. Torres, Natalia Gutierrez

**Affiliations:** ^1^ Julius Kühn-Institut (JKI), Federal Research Centre for Cultivated Plants, Institute for Resistance Research and Stress Tolerance, Sanitz, Germany; ^2^ Área de Mejora Vegetal y Biotecnología, Andalusian Institute of Agricultural and Fisheries Research and Training (IFAPA), Centro Alameda del Obispo, Córdoba, Spain

**Keywords:** faba bean, drought stress, phenotyping, physiological traits, senescence, genetic diversity, heritability, yield

## Abstract

Faba bean is an important protein crop for food and feed worldwide and provides a range of advantages in crop rotations. Its limited use in modern agriculture is mainly due to the high fluctuations in yield. A well known limiting factor in most legumes, and particularly in faba bean, is the high sensitivity to water shortage, which is further aggravated by climate change. The present study was undertaken to exploit the genetic variation in drought stress response in a faba bean collection of 100 accessions with diverse origins and to assess selection criteria for identifying drought tolerant genotypes. Physiological, phenological and yield related traits evaluated under drought or water-sufficient conditions responded significantly to the end-terminated drought stress. Comparison of yield relations showed the advantage of using a stress tolerance index (STI) to identify genotypes combining high yield potential with high stress yield. With regard to physiological traits, SPAD (chlorophyll content) values were significantly related to yield as well as to STI, while the other traits also contributed to different extents to variation in yield formation. Among the yield related traits, seeds per plant proved to be the most important trait followed by pods per plant. Interestingly, the eight genotypes with the best STI performance use different strategies to cope with drought stress.

## Introduction

Grain legumes are a major source of plant protein for food and feed worldwide and provide multiple advantages in crop rotations regarding soil fertility, plant health and sustainability. Faba bean (*Vicia faba* L.) is one of the most widely distributed crops, being grown both as a grain (pulse) and green-manure legume (Jensen et al., 2010). With a production varying between 4.5 and 5.5 Mt in the last decade, faba bean ranks 6th in terms of world production of pulse crops (FAOSTAT, 2022). China is the largest producer followed by Ethiopia, the United Kingdom, Australia, France and Germany. In the last 70 years, there has been more than 50% decline in the global cropping area of the faba bean, which is mainly caused by poor yield stability owing to climate variability, diseases, weeds and other pests, which represent the major constraints of faba bean production.

Faba bean yield fluctuations are likely to increase with the predicted climate change in this century. Increasing atmospheric CO2 concentrations will change surface temperatures and precipitation patterns, causing more periods of extreme precipitation and drought (Farooq et al., 2014). Therefore, drought stress has become one of the most uncontrollable and unpredictable agricultural challenges and is today one of the major yield limiting factors in grain legume crops (Swann et al., 2016; Nadeem et al., 2019). Improving drought tolerance is a key strategy to enhance performance and stability of yield in these crops. Nevertheless, the complexity of the trait, the lack of efficient selection protocols and the mixed faba bean breeding system pose major challenges for effective implementation in plant breeding programs (Muktadir et al., 2020).

Legumes respond to drought with morphological, physiological and biochemical changes in roots, stems and leaves (Shakeel et al., 2011). Drought stress results in many interactive modifications including changes in the expression of drought-resistance genes, the synthesis of hormones, the overproduction of reactive oxygen species and the osmotic adjustments through active ions or organic compound such as proline and carbohydrates (Farooq et al., 2012; Kaur and Asthir, 2015; Wahab et al., 2022). Plants minimize water loss by closing stomata and reducing light absorbance, canopy leaf area and peaked water absorption.

The extent and type of responses will depend on the intensity and length of the stress, but in all cases grain yield is substantially reduced. A meta-analysis of legume yield responses to drought under field conditions from 1980 to 2014 revealed a yield reduction of 40% following a 65% reduction in water availability, with cultivar and environmental factors being important cofactors (Daryanto et al., 2015). The study confirmed, that legumes have a high yield potential, but respond in a sensitive way to water shortage (Müller et al., 1985; Grashoff, 1990; Khan et al., 2010; Torres et al., 2010; Redden et al., 2014; Muktadir et al., 2020). Faba bean is more sensitive to drought than other field crops and largely exceeds other grain legumes such as common bean, pea and chickpea (McDonald and Paulsen, 1997; Amede and Schubert, 2003; Manning et al., 2020).

Drought impairs grain legume yield at all growth stages. Germination, leaf area and photosynthetic activity are significantly reduced (Nadeem et al., 2019). However, the most sensitive period is the reproductive stage where drought stress leads to earlier flowering, and ultimately, reduced pod and grain set (Plies-Balzer et al., 1995; Khan et al., 2007; Alghamdi et al., 2015). Against this background, improvement of drought tolerance is an important faba bean breeding goal. Understanding of drought response patterns and associated traits are key factors for achieving higher yield stability under stress conditions. However, compared to other crops, improvement of productivity under drought stress has rarely been included in faba bean breeding programmes. Breeding progress has been relatively slow so far due to the limited number of genotypes included in the studies (Abdelmula et al., 1999), the low heritability of advantageous traits and the lack of efficient and reproducible screening methods (Stoddard et al., 2006; Muktadir et al., 2020). However, a wide genotypic variation in faba bean water stress response has been reported (Link et al., 1999; Amede et al., 2001; Ricciardi et al., 2001; Khan et al., 2007), indicating a high potential for breeding in drought-prone environments. To exploit this genetic variation, the application of an accurate and relevant phenotyping method plays a key role for the selection of drought-resilient genotypes and for the dissection and genetic analysis of a complex trait such as the adaptive response of crops to drought (Tuberosa, 2012).

Phenotypic attributes are the most frequently used criteria used for identifying drought-tolerant genotypes. In legumes, selection for drought tolerance based on highly heritable morphological and phenological traits, together with physiological attributes such as the accumulation of proline or soluble sugars, has proven highly successful for screening genotypes under limited water supply (Lafitte et al., 2003; Richards, 2006; Stoddard et al., 2006; Annicchiarico and Iannucci, 2008; Alderfasi and Alghamdi, 2010; Ammar et al., 2015; Ali et al., 2016). However, phenotyping under field conditions is time- and labour-consuming, and the reproducibility of water stress conditions is often poor, because of variations in the timing of onset, duration and severity of the drought (Khan et al., 2007). The recent use of a controlled water supply combined with rainout shelters reduces these uncertainties and generates results that are more accurate. These tools allow phenotyping of large field populations under conditions of adequate light intensity and quality, avoiding the effects of unpredictable rainfall patterns.

Direct selection for grain yield under drought conditions often proves inefficient to identify stress-tolerant genotypes. For this reason, several selection indices have been developed to evaluate yield stability based on grain yield under normal and stress conditions (reviewed in Sofi et al., 2018). Among these, the stress tolerance index (STI) defined by Fernandez (1992) has been consistently correlated with other indexes (Farshadfar et al., 2013; Mansour et al., 2021; Sharifi et al., 2021; Memari et al., 2022), indicating that it can be used as an alternative to select drought tolerant genotypes with high yield performance in stress and non-stress conditions.

Considering the scarce number of studies so far reported and the limited number of faba bean genotypes used, breeding progress for drought stress tolerance in faba bean has been relatively slow. The goal of the present study was to exploit the genetic variation of a wider collection of 100 faba bean accessions with diverse origins to assess the selection criteria for identifying drought tolerant genotypes and to dissect a complex trait such as the adaptive response to drought. Thus, our main aims were: (i) to evaluate the performance of this faba bean collection, one of the largest screenings reported so far, under water-sufficient and drought stress conditions and (ii) to investigate the relevance of morphological and phenological traits with respect to yield and yield stability in water stress conditions in an effort to identify and to improve selection efficiency under drought stress environments.

## Materials and methods

### Plant material and experimental design

The test set included 100 faba bean inbred lines of diverse origins, which were grown in 2019 and 2020 in Groß Lüsewitz (north-eastern Germany). The location is characterized by an annual mean temperature of 8.3°C. Soil type is a slightly loamy sand with a pH of 5.7.

The worldwide diversity panel includes genetic stocks aiming at gathering a wide range of genetic diversity from diverse geographical origins: Africa (8 accessions), North and South America (2), Asia (27) and Europe (39). The remaining 24 accessions were provided by the International Center for Agricultural Research in the Dry Areas (ICARDA), but their exact origin is unknown. Europe, with 9 countries, is the most represented geographical area, followed by Asia, Africa and America (7, 4 and 2 countries, respectively). Spain accounts for the highest number of accessions (23). The diversity panel was built in collaboration with the public institutes ICARDA, the Andalusian Institute of Agricultural and Fisheries Research and Training (IFAPA) and the French National Institute for Agricultural Research (INRA). Prior to the genotyping analysis, all the Spanish lines had been self-pollinated for at least four generations. The remaining accessions were purified for two generations by single seed descent (SSD) in insect-proof cages. A detailed description of this collection is provided in **Supplementary Table S1**.

**Figure 1:**
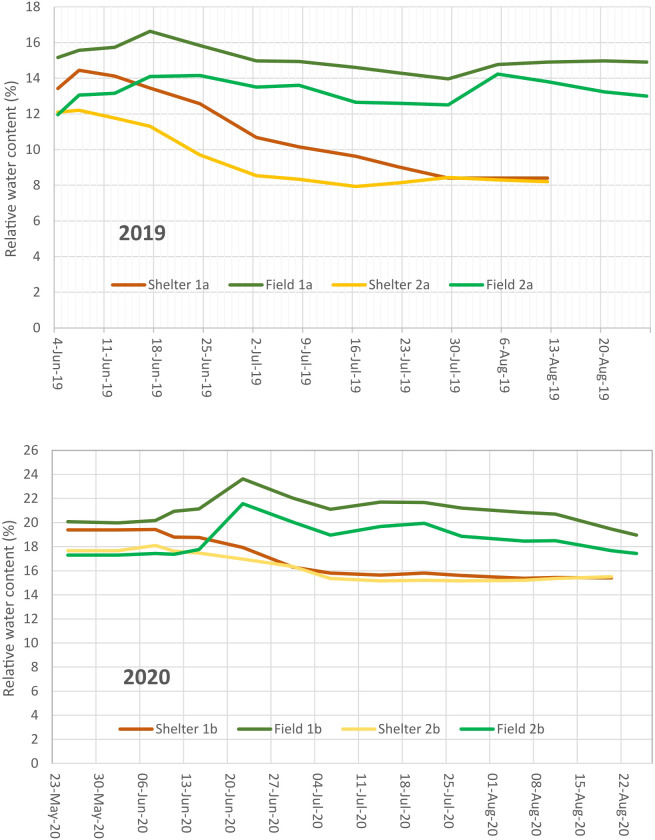
Soil water content of the experimental areas measured by TDR probes in about 0.40 m depth for the first and second experimental year.

The experimental set up was a randomized complete-block design with 4 replications in both treatments. Drought stress treatment was performed in two mobile rainout shelters, with two blocks in each. The same design was applied in the open field control treatment. Plants were grown in single row plots of 1.2 m length with 14 plants each (one seed per hill) and the distance between plots was 0.5 m. Additional water was applied by drip irrigation (**Supplementary Figure S1**). Irrigation has been scheduled in the range of 60 - 70% of field capacity of the soil, determined over winter after excessive rainfall. Water content in the soil was assessed by time-domain reflectance (TDR) probes in about 0.40 m depth (**Figure 1**). Irrigation in the stress treatment was stopped when about 30% of the genotypes had started flowering. During rainfall the shelter covered the plots. An end-terminated drought stress was applied. The key data of the experiments are explained in **Supplementary Table S2**.

### Weather conditions

The mean temperature, the Crop Heat Unit (CHU) based heat sums and the precipitation conditions during the vegetative period in the two years of the experiment are summarized in **Table 1**. The experimental year 2019 was moderate with respect to rainfall but warm with an early heat period in June. The year 2020 was cooler with slightly more rainfall and a late heat period in August, when faba beans were partly harvested already. This resulted in a more intensive drought stress in 2019 compared to 2020.

**Table 1:** Weather conditions during the vegetative period in 2019 and 2020 at Gross Lüsewitz.

	Mean Temperature (°C)		CHU* heat sums (°C)		Precipitation (mm)	
	2019	2020	2019	2020	2019	2020
**April**	9.2	8.4	259	188	23.9	10.9
**May**	10.9	10.9	311	279	33.1	20.5
**June**	19.1	16.6	665	579	87.4	131.2
**July**	17.7	16.0	644	571	79.7	62.1
**August**	18.5	19.4	685	708	55.7	82.2
**Sum**			2564	2325	279.8	306.9

*Crop Heat Units.

### Measurements of traits

Four physiological traits were assessed in the youngest fully-developed leaves from the main stem of the inner 10 plants of each plot. Two leaf samples mixed from 5 plants per plot each were taken about 4 weeks after onset of stress, according to the stress development, for determining the content of free proline (PRO) and total soluble sugars (TSS). Samples were immediately frozen in liquid nitrogen. PRO was analyzed as described by Bates et al. (1973) and TSS was estimated by the anthrone reagent method according to Yemm and Willis (1954). SPAD measurements as an indirect parameter for chlorophyll content were carried out with a Chlorophyll Meter SPAD 502 Plus (Konica Minolta) at the beginning of stress treatment (SPAD1) and in the same time slot of TSS and PRO sampling (SPAD2). The mean value of 30 clippings per plot was used. The difference between these SPAD values, DiffSPAD (DiffSPAD = SPAD1 – SPAD2), was calculated to standardize the possible differences in chlorophyll content among genotypes and to obtain a more accurate measure of chlorophyll degradation (senescence). Two phenological traits were analyzed: end of flowering (EF), recorded as the days after sowing when no open flowers were visible any more in the plot (BBCH 69); and maturity date (MAT), defined as the date when more than 90% of the pods have ripened (BBCH 89) following the extended BBCH Code (Hack et al., 1992). Moreover, five morphological traits were recorded: plant height in cm (PH); number of pods per plant (PP); number of seeds per plant (SP); hundred seed weight (HSW) in grams; and plot yield (PY) in kg. Plant height was measured close to maturity (BBCH 80-85) from the ground to the tip of 10 inner plants per plot. At the end of the vegetative period, 10 single plants per plot were harvested by hand and pods and seeds were counted. Dry matter content was determined at 105°C. PY and HSW are given on a basis of 86% dry matter.

### Data analyses

Statistical analyses were performed by SAS 9.4 (SAS Institute, USA). Analysis of variance (ANOVA) used a linear mixed model with year as a random effect. Means are shown as LSmeans. Minimum variance quadratic unbiased estimators (MIVQUEs) of variance components were used to calculate broad sense heritability h² according to Becker (2011). The response of the accessions to drought stress was calculated as relative yield of the drought stress treatment:


Yrel=PY_ DS×100/PY_C

with PY_DS as plot yield of the drought stress and PY_C as plot yield of control treatment.

The Stress Tolerance Index (STI) was calculated for the yield measured in both treatments (control and drought) according to Fernandez (1992) as follows:


$$STI=PY\_C\times PY\_DS/{\left(\bar{ϒ}\_C\right)}^{2}$$

with PY _ C as plot yield of control, PY_DS as plot yield of the drought stress treatment and $\bar{ϒ}\_C$ as mean yield of the control environment.

Pearson’s correlations between STI, PY_C and PY_DS data were calculated in order to check for possible dependencies between the performance of the accessions under control conditions and the strength of response to drought stress. A principal component analysis (PCA) was calculated based on correlations using JMP Genomics 9 (SAS Institute, USA). The results were represented using a biplot that combines both the principal component scores and the loading vectors in a single plot. In addition, a cluster analysis of observations was performed to find subgroups within phenotypic data in control and drought conditions by means of the K-means clustering method (Hartigan and Wong, 1979). The data were scaled to make variables comparable. The average silhouette approach was used to determine the optimal number of clusters. K-means clustering was performed using the built-in k-means libraries of R suite and similarities were measured by squared Euclidean distance to classify all the accessions into groups (R Core Team, 2022). The results were visualised by means of the ‘fviz_cluster’ function in the ‘factoextra’ R-package (Kassambara and Mundt, 2020).

## Results

### Statistics of the data

Descriptive statistics and analysis of variance (ANOVA) of the traits recorded over two years is shown in **Table 2**. The table includes the mean, range and Least Significant Difference (LSD) values together with the heritability (h²) of the traits in both conditions. LSDs of all the traits studied in the 100 faba bean genotypes in control and drought stress conditions are provided in **Supplementary Table S3**. Most of the parameters displayed a wide range of differences among the treatments. A significant effect of genotype and drought stress treatment as well as an interaction between them could be observed for all traits.

**Table 2:** Descriptive statistics and analysis of variance (ANOVA) of the traits over both years.

Trait		Control	Stress	Unit	F values				Heritability (h²)	
					Genotype	Treatment	G x T	Year	Control	Stress
Soluble sugars (TSS)	Range LSD	1117.6 769.5 - 1709.4 587.1	1387.9 998.2 - 2242.4 659.9	µmol/ g DM	7.44 ***	339.79 ***	1.86 ***	196.73 *** 2019>2020	0.56	0.66
Free proline (PRO)	Range LSD	2.29 1.50 - 6.55 1.20	8.45 1.79 - 57.02 26.75	µmol/ g DM	2.93 ***	171.09 ***	2.57 ***	128.78 *** 2019>2020 stress only	0.63	0.3
SPAD 2	Range LSD	42.8 29.2 - 57.4 8.5	24.0 11.5 - 51.3 9.8	SPAD Units	22.57 ***	6780.40 ***	9.30 ***	208.32 *** 2020>2019	0.73	0.89
Difference SPAD (DiffSPAD)	=Senescence Range LSD	-5.0 -15.1 - 5.2 10.1	15.5 -6.8 - 25.2 11.0	SPAD Units	11..56 ***	6100.08 ***	8.30 ***	54.74 *** 2019>2020	0.38	0.86
End of flowering (EF)	Range LSD	97,0 85.5-118.6 15.7	86.3 82.0-100.3 11.2	DAS	8.13 ***	1169.23***	2.33***	230.27*** 2019>2020	0.73	0.44
Maturity (MAT)	Range LSD	127.4 118.0-143.0 10.1	115.1 105.9 - 126.5 9.2	DAS	9.28 ***	2404.57 ***	1.51 **	54.41 *** 2019>2020 C 2020>2019 S	0.75	0.68
Plant height (PH)	Range LSD	64.6 42.1 - 102.3 18.5	56.5 37.1 - 71.6 12.2	cm	17.19 ***	359.77 ***	3.19 ***	170.93 *** 2020>2019 control only	0.87	0.82
Pods per plant (PP)	Range LSD	10.8 4.5-27.1 6.9	6.4 3.5 - 12.0 3.2	number	19.11 ***	1124.49***	5.22***	1.59*** 2020>2019 control only	0.89	0.88
Seeds per plant (SP)	Range LSD	24.9 8.9 - 77.9 17.3	14.2 5.8 - 27.0 7.5	number	21.3 ***	1039.57 ***	6.86 ***	1.60 *** 2020>2019	0.9	0.85
Hundred seed weight (HSW)	Range LSD	66.93 30.27 - 115.38 23.67	60.44 26.07 - 103.24 16.28	g/100 seeds	28.6 ***	133.13 ***	1.39 **	420.52 *** 2019>2020	0.83	0.91
Plot yield (PY)	Range LSD	0.155 0.047 - 0.321 0.119	0.083 0.031 - 0.117 0.054	kg/plot	9.19 ***	1003.33 ***	3.26 ***	5.0 * 2019>2020	0.83	0.65
Stress tolerance index (STI)	Range LSD	0.564 0.074 - 1.378 0.048			4.94 ***			1.38 n.s.	0.80	

***, ** and * are significant for α ≤ 0.1, 1 and 5%, respectively, n.s., not significant; LSD, Least Significant Difference; DAS, Days after sowing.

**Figure 2:**
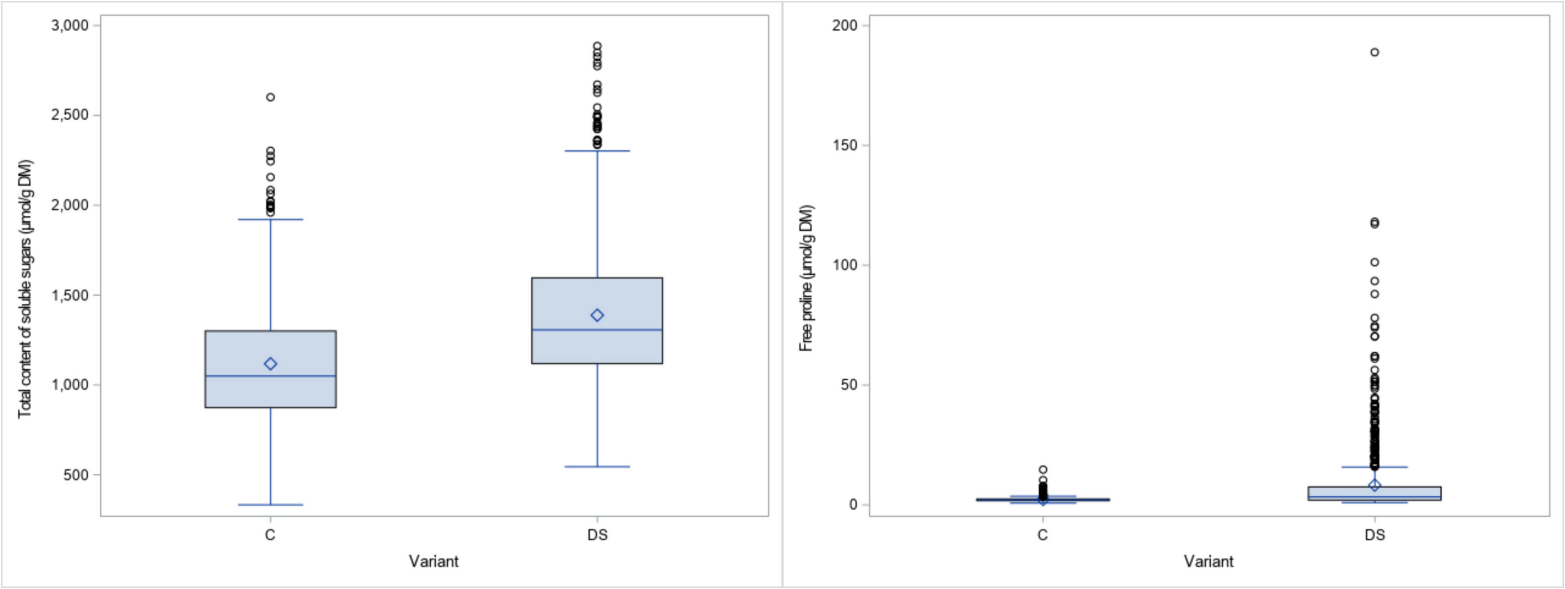
Boxplots for total content of soluble sugars (TSS) and free proline (PRO) for control (C) and drought stress (DS) treatment.

**Figure 3:**
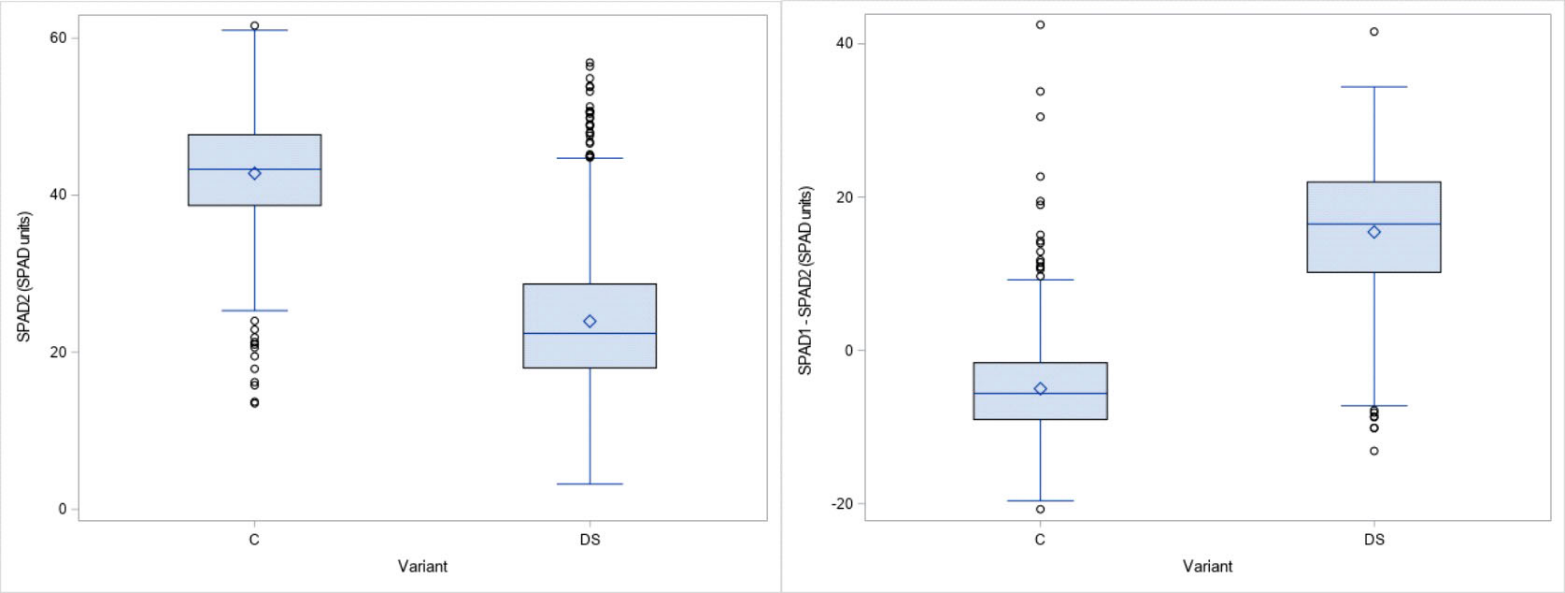
Chlorophyll content by SPAD Meter about 4 weeks after onset of drought stress (SPAD 2) and difference to SPAD 1 values (DiffSPAD) for control (C) and drought stress (DS) treatment.

**Figure 4:**
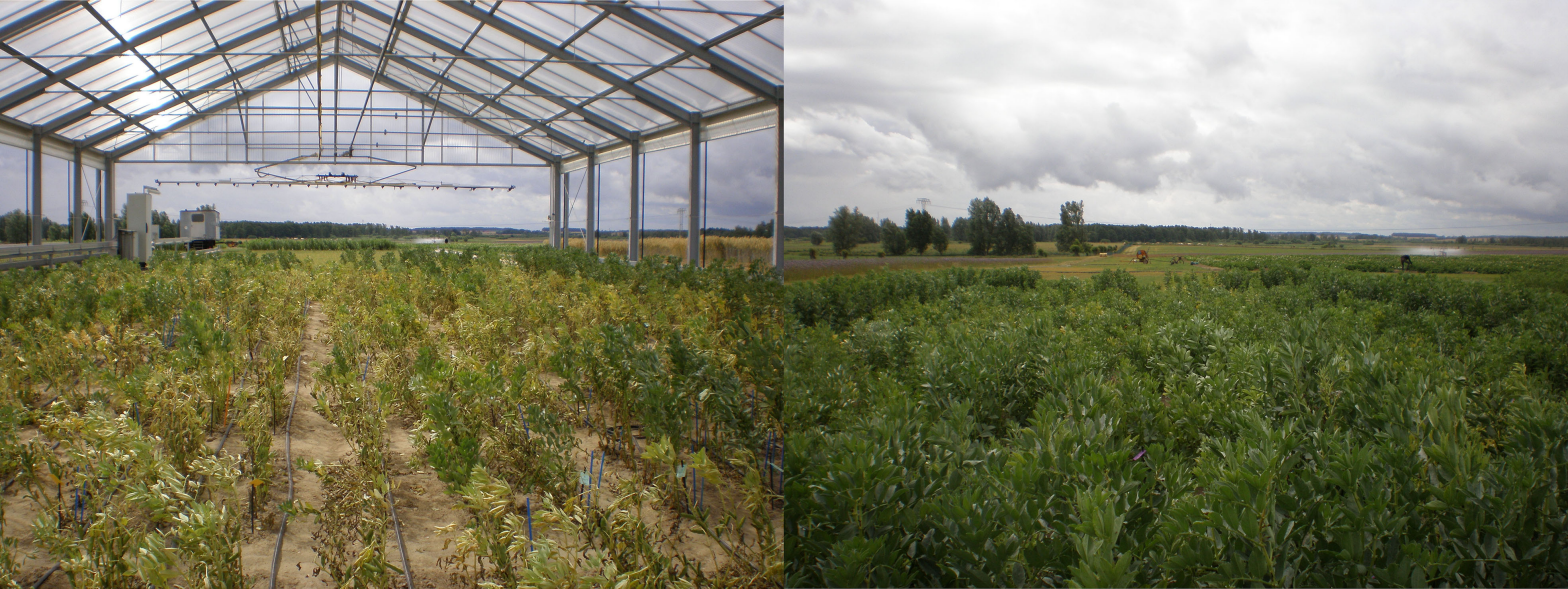
Visible signs of early senescence under drought stress in rain out-shelter (left side) compared to the control (right side) at the beginning of July 2019.

The results obtained for the physiological traits showed clear differences between treatments. TSS and PRO were measured when drought stress was fully developed and senescence started in the stress treatment. As expected, both parameters increased significantly (24% and 269% respectively) under drought stress (**Figure 2**). The significant interaction between genotype x treatment indicates a different response of genotypes to drought stress (**Table 2**). The leaf chlorophyll concentration, assessed as SPAD values, showed a marked decrease (44%) after 4 weeks of drought stress compared to the control (SPAD2 and DiffSPAD, **Figure 3**), which was clearly visible (**Figure 4**). This was confirmed by the difference between the two SPAD values (DiffSPAD = SPAD1 – SPAD2) which minimizes the effects of variability in chlorophyll content among genotypes and stands for the degree of senescence. Whereas in the control treatment values were increasing until full flowering (negative difference), chlorophyll content was already decreasing in the stress treatment (positive difference).

**Figure 5:**
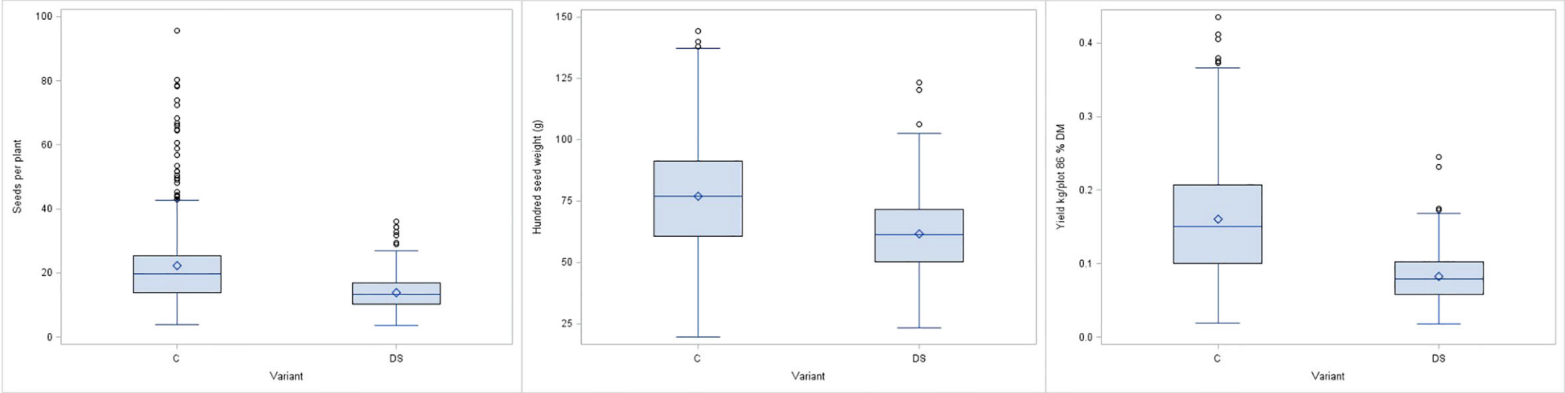
Boxplots for seeds per plant (SP), hundred seed weight (HSW) and plot yield (PY) for control (C) and drought stress (DS) treatment.

For all the phenological and morphological traits, the mean values also decreased under drought stress conditions with values ranging from 9.7% for MAT to 11.3% for EF (**Table 2**). PH decreased by 12.5% and the taller forms were more affected than the shorter types (data not shown). In contrast, strict declines were observed for the remaining yield related traits PP, SP and PY. Mean PY value under drought stress was significantly reduced by 46.4%, while PP and SP were reduced by 40.8% and 42.8%, respectively. Similarly, HSW values were lower, compared to the control, but the mean decrease of 9.7% was not as high as that of the other yield components (**Figure 5**). A significant interaction between genotypes and drought stress treatment was detected.

Heritability computed across the two water regimes (control vs. drought) ranged from 0.3 (PRO) to 0.91 (HSW), both in stress and non-stress conditions (**Table 2**). Heritability was similarly high (> 0.82) under both conditions for the yield related traits PH, PP, SP and HSW. Moderate to high heritability was recorded for the physiological traits TSS (0.56-0.66), MAT (0.75-0.68), SPAD2 (0.73-0.89), DiffSPAD (0.38-0.86) and PY (0.83-0.65) in both conditions. The most contrasting heritability values between water regimes were found in EF (0.73-0.44) and PRO (0.63-0.3).

### Yield relationships and genotypes

**Figure 6:**
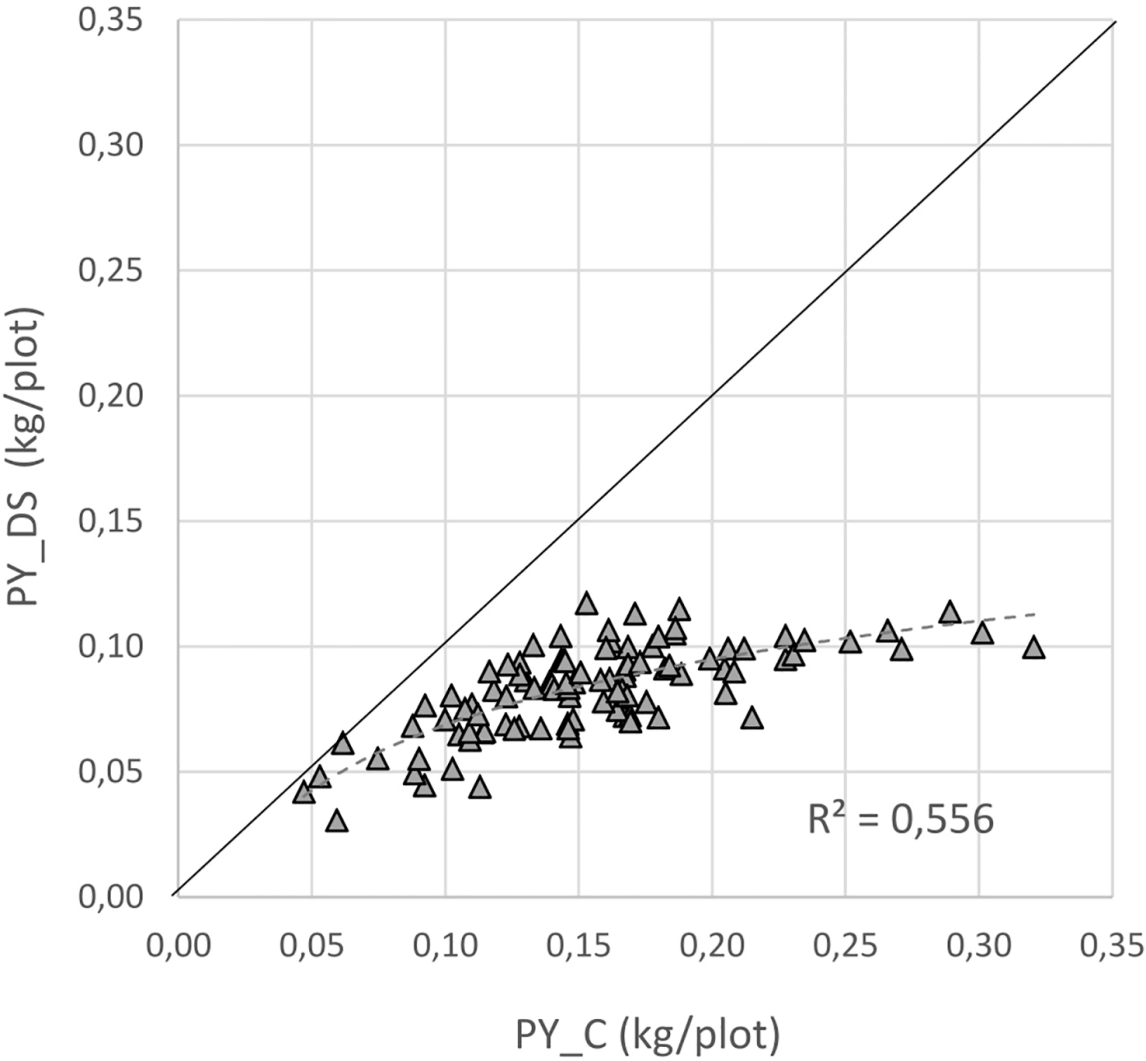
Relation between yield under drought stress (PY_DS) and yield in the control (PY_C); the solid black line stands for 100% yield stability.

**Figure 7:**
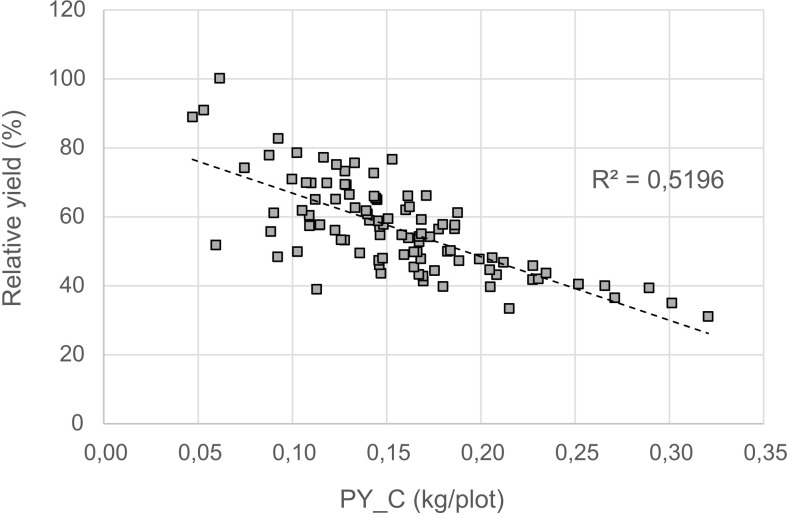
Relationship between relative yield and yield in the control (PY_C).

**Figure 8:**
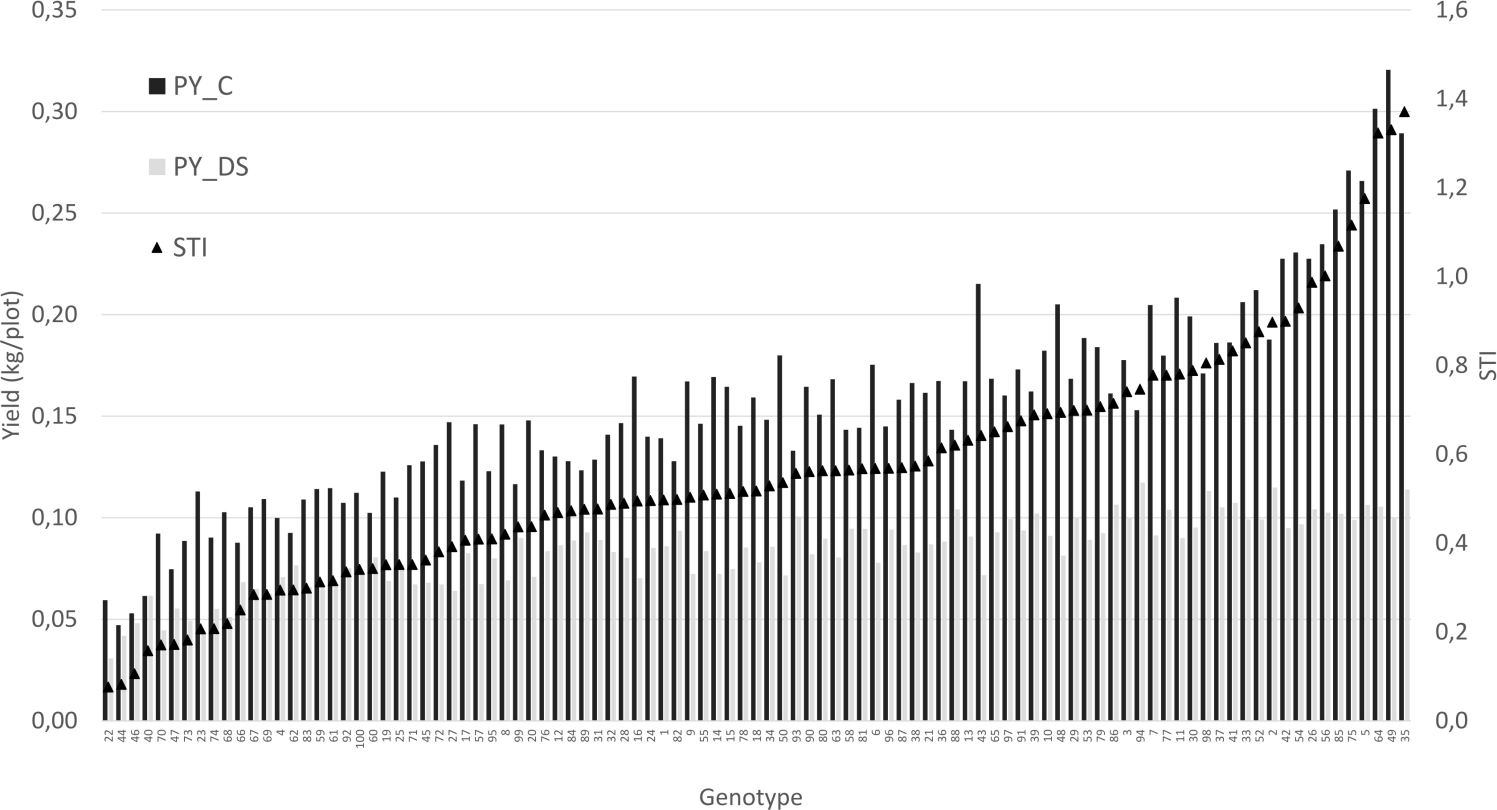
Genotypes of the test set ordered according to their STI value. PY_C: control yield. PY_DS: drought stress yield.

For a better understanding of the relationship between yield under control and stress conditions, a regression analysis was conducted. This relationship provides a benchmark for identifying the most drought tolerant genotypes (**Figure 6**). A positive correlation was detected between the two parameters (r = 0.75), whereby the high yielding genotypes were more affected by drought stress than the low yielding ones.The non-linear regression line shows that stress yield may not exceed a distinct level predefined by the stress environment. A correlation coefficient of -0.72 (**Figure 7**) revealed the negative and relatively close correlation between the yield under controlled conditions (standing for yield potential) and the relative yield (standing for yield stability). The figure clearly depicts that selection of genotypes with a high relative yield in most cases leads to rather low yielding genotypes. For this reason, a stress tolerance index (STI) based on seed yield (PY) was calculated for each genotype in response to severe drought and well-watered conditions, in order to identify genotypes producing high yields under both conditions. The variability of this index is shown in **Figure 8**. By using STI as target parameter, it was possible to identify genotypes that combine a high yield potential with a reasonable stress yield in this faba bean assortment. Accession ID and origin of the eight most drought-tolerant genotypes (5, 26, 35, 49, 56, 64, 75 and 85), according to STI are given in **Table 3**.

**Table 3:** STI value, ID and origin of the eight best genotypes.

STI value	Genotype	Accession ID	Accession	Origin
1.371	35	EUC_VF_077	1272-1	RUS
1.330	49	EUC_VF_130	INRA 2394	DEU
1.323	64	EUC_VF_194	INRA 612	EGY
1.176	5	EUC_VF_ 009	359-1	TUR
1.116	75	EUC_VF_ 303	IG 72242	CHN
1.068	85	EUC_VF_336	Misr 3	EGY
1.001	56	EUC_VF_174	1248-3	IRN
0.987	26	EUC_VF_064	1157-1	ESP

**Figure 9:**
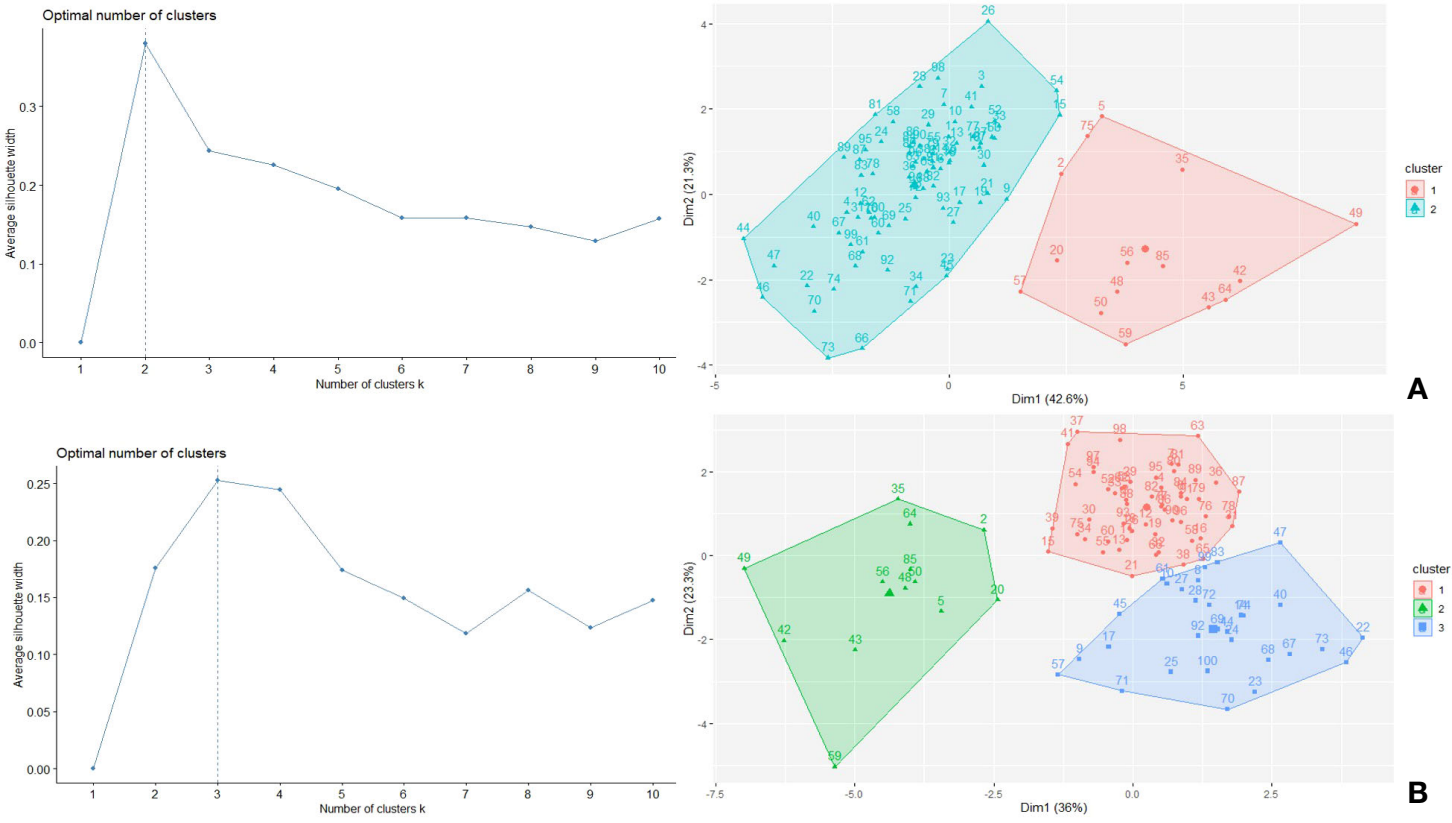
Optimum number of clusters and clustering of the 100 faba bean accessions under control **(A)** and drought **(B)** conditions.

The K-means cluster analysis using the morphological, phenological and physiological faba bean data and the STI index in control and drought conditions classified the accessions into groups based on their dissimilarities (**Figure 9**). For control conditions, two clusters were identified: cluster 1 (15 genotypes) showed highest STI value and highest average values for all traits (except for HSW), containing six of the eight best performing STI genotypes (5, 35, 56, 64, 75 and 85), while cluster 2 (84 genotypes) had the maximum average value for HSW and contained the remaining genotypes 26 and 49 (**Table 4**). Under drought conditions, the genotypes were classified in three clusters: cluster 1 (56 genotypes), cluster 2 (13 genotypes) and cluster 3 (30 genotypes). Cluster 2 showed the best average value for PRO, SPAD2 (and correspondingly low DiffSPAD), MAT, PH, PP, SP, PY and STI, while cluster 1 had a similar yield but an intermediate value for STI and cluster 3 had the lower yield and STI mean values but the highest ones for TSS, EF and HWS (**Table 4**). Again, six of the best STI genotypes (accessions 5, 35, 49, 56, 64 and 85) were included in cluster 2 while cluster 1 contained the other two accessions (26 and 75). According to cluster analysis, genotypes from cluster 2 were superior regarding the STI index and yield related traits, so cultivation of these genotypes would be recommended under drought stress conditions.

**Table 4:** Average value based on physiological, phenological and morphological and the STI index under control (C) and drought stress conditions (DS) after K-means cluster analysis.

Cluster	TSS_C	PRO_C	SPAD2_C	DiffSPAD_C	EF_C	MAT_C	PH_C	PP_C	SP_C	HSW_C	PY_C	STI
1 (15)*	1212.53	2.95	44.66	-5.51	102.27	132.62	84.89	17.85	44.33	51.04	0.21	0.82
**2 (84)**	1099.55	2.13	42.45	-4.90	96.01	126.39	60.48	9.46	21.28	69.97	0.14	0.51
Cluster	TSS_DS	PRO_DS	SPAD2_DS	DiffSPAD_DS	EF_DS	MAT_DS	PH_DS	PP_DS	SP_DS	HSW_DS	PY_DS	STI
**1 (56)**	1276.416	6.671	21.529	18.183	84.221	112.875	54.782	6.405	14.869	62.408	0.091	0.612
**2 (13)**	1310.345	17.398	37.205	2.385	88.485	119.037	65.862	9.148	20.596	45.726	0.092	0.899
**3 (30)**	1634.117	6.995	22.449	16.267	88.961	117.319	55.341	5.157	10.324	63.628	0.065	0.321

* Number of genotypes in each cluster.

### Correlations and principal component analysis (PCA)

A correlation analysis was performed to evaluate the relationships among morphological, phenological and physiological traits as well as their association with PY and STI. As mentioned above, the correlations of plot yield under control (PY_C) and drought stress conditions (PY_DS) with STI are similar and highly significant, with PY_C having the greater impact on STI (**Table 5**). The most yield influencing factors were SP and PP, although physiological traits such as chlorophyll content (SPAD values), PRO and TSS together with phenological traits such as EF and MAT, also contributed significantly to the respective yield and to the STI values.

**Table 5:** Correlation matrix with Pearson’s correlation coefficient r for all relevant traits under control (above) and drought stress conditions (down).

Trait	TSS _C	PRO_C	SPAD2_C	DiffSPAD_C	EF_C	MAT_C	PH_C	PP_C	SP_C	HSW_C	PY_C	STI
**TSS_C**	1	-0.097	**-0.290**	**0.482**	**0.385**	**0.276**	**0.284**	0.118	0.026	-0.219	-0.210	**-0.256**
**PRO_C**		1	**0.260**	-0.164	0.185	**0.357**	**0.560**	**0.562**	**0.616**	**-0.358**	**0.421**	**0.352**
**SPAD2_C**			1	**-0.812**	0.035	**0.372**	**0.263**	**0.310**	**0.345**	0.129	**0.509**	**0.474**
**DiffSPAD_C**				1	-0.004	**-0.259**	-0.163	**-0.261**	**-0.267**	-0.200	**-0.474**	**-0.448**
**EF_C**					1	**0.694**	**0.408**	**0.255**	0.132	-0.106	-0.017	-0.105
**MAT_C**						1	**0.619**	**0.444**	**0.377**	-0.007	**0.374**	**0.275**
**PH_C**							1	**0.783**	**0.767**	**-0.340**	**0.590**	**0.484**
**PP_C**								1	**0.925**	**-0.515**	**0.663**	**0.566**
**SP_C**									1	**-0.499**	**0.737**	**0.650**
**HSW_C**										1	0.153	0.201
**PY_C**											1	**0.961**
**Trait**	**TSS _DS**	**PRO_DS**	**SPAD2_DS**	**DiffSPAD_DS**	**EF_DS**	**MAT_DS**	**PH_DS**	**PP_DS**	**SP_DS**	**HSW_DS**	**PY_DS**	**STI**
**TSS_DS**	1	0.217	0.197	0.187	**0.267**	**0.333**	0.035	**-0.293**	**-0.414**	0.123	**-0.441**	**-0.410**
**PRO_DS**		1	**0.510**	**-0.459**	0.100	**0.343**	**0.279**	0.171	**0.254**	-0.052	0.187	**0.369**
**SPAD2_DS**			1	**-0.905**	**0.282**	**0.558**	**0.442**	**0.423**	**0.478**	**-0.325**	0.137	**0.373**
**DiffSPAD_DS**				1	**-0.269**	**-0.504**	**-0.392**	**-0.420**	**-0.432**	**0.327**	-0.068	**-0.317**
**EF_DS**					1	**0.680**	**0.253**	-0.014	-0.172	-0.029	**-0.405**	**-0.250**
**MAT_DS**						1	**0.393**	0.076	-0.090	0.032	-0.182	0.023
**PH_DS**							1	**0.500**	**0.398**	-0.195	0.206	**0.381**
**PP_DS**								1	**0.819**	**-0.626**	**0.332**	**0.429**
**SP_DS**									1	**-0.651**	**0.499**	**0.523**
**HSW_DS**										1	**0.268**	0.129
**PY_DS**											1	**0.836**

Numbers in bold are significant for α ≤ 5%. PRO, content of free proline; TSS, total content of soluble sugars; EF, end of flowering; MAT, maturity; PH, plant height; PP, pods per plant; SP, seeds per plant; HSW, hundred seeds weight; PY, plot yield; STI, stress tolerance index; C, control; DS, drought stress.

Under control conditions, PH was significantly correlated with most traits, particularly PP and SP, highlighting their contribution to yield. Strong positive correlations were also observed for PP and SP with STI, PY and PRO. Interestingly, HSW showed a strong negative correlation with PP and SP. Under drought stress conditions, DiffSPAD showed a significant negative correlation with all traits except HSW. SPAD2 showed strong positive correlation with MAT and PRO and lower but still significant correlation with PH, PP and SP, which showed positive inter-correlations. In control conditions, all measured yield-related traits (PY, PP, and SP) were associated positively except HSW, indicating that they could simultaneously improve drought tolerance at the expense of seed weight. Correlations were stronger in the control treatment in nearly all cases with the exception of EF and TSS (**Table 5**).

**Figure 10:**
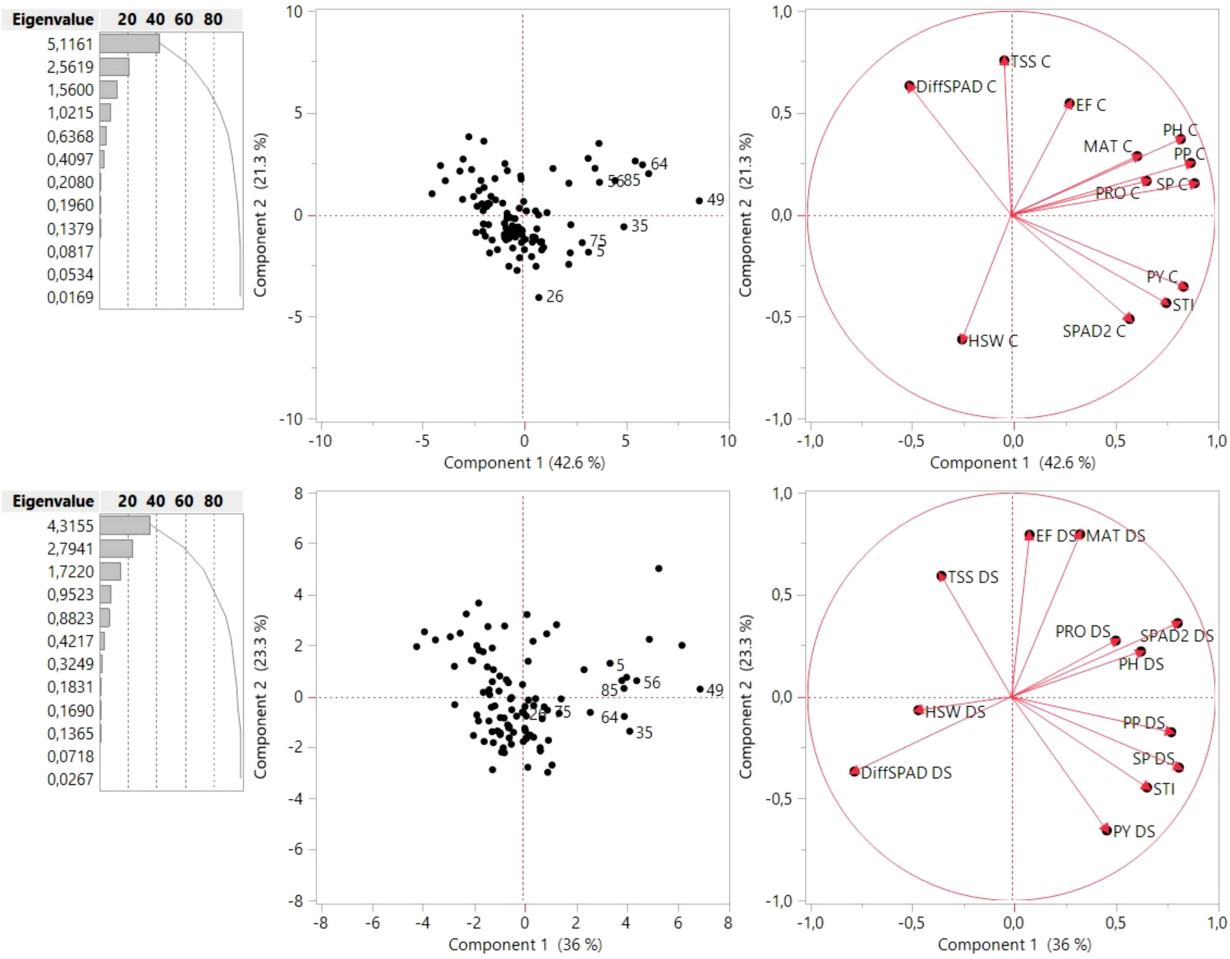
PCA based on all Least Squares (LS) means of the 100 genotypes for the respective traits assessed under control (above) and drought stress conditions (down). Points with numbers are the eight best genotypes according to STI (see ). The lines indicate eigenvectors representing the strength (length of the vector) and the direction of the parameter correlation relative to the first two principal componente (PC1 and PC2). TSS, total content of soluble sugars; PRO, free proline content; SPAD, measures of chlorophyll content 4 weeks after onset of stress (SPAD 2) and differences in chlorophyll content (DiffSPAD); EF, end of flowering; MAT, maturity; PH, plant height; PP, pods per plant; SP, seeds per plant; HSW, hundred seed weight; PY, plot yield; STI, stress tolerance index; C, control, DS, drought stress.

In order to determine the most contributing traits to yield under both conditions, a principal component analysis (PCA) was performed (**Figure 10**). Results showed that the first and second component (PC1 and PC2) represented 63.9% of the variance observed in control conditions and 59.3% of the variance in the drought stress treatment. Biplots for control and drought conditions were constructed from PC1 (42.6% and 36% variation explained, respectively) and PC2 (21.3% and 23.3% variation explained, respectively) with the distribution of the 100 genotypes, the morphological, phenological and physiological traits and STI. The PCA matrix revealed that the genotypes assayed followed different strategies with respect to seed set, seed size or physiological parameters (**Figure 10**). In the control condition, the PC1 axis was primarily associated with SP and PP, followed by PH and PRO, while TSS and differences in chlorophyll content (DiffSPAD) were the most contributing factors for the dispersion of the genotypes along PC2. In the drought stress treatment, chlorophyll content (SPAD2), followed by SP and PP had the largest contribution to PC1, while EF and MAT mainly contributed to PC2. The nearly 180° angle between SPAD2 and DiffSPAD indicated a strong negative association, as expected. PCA biplot analysis further demonstrates that the eight superior genotypes according to STI follow different strategies with respect to phenological, physiological and yield components. Thus, in drought conditions the genotypes 26, 35, 64 and 75 showed a higher number of pods (PP_DS), seeds per plant (SP_DS) and yield (PY_DS) as well as a higher STI value, but low total soluble sugar content (TSS_DS). On the other hand, genotypes 5, 49, 56 and 85 showed higher values for plant height (PH_DS) and the physiological traits SPAD2 and PRO, together with a low hundred seed weight (HSW_DS).

## Discussion

In this study, a faba bean collection of 100 accessions was used to assess the relevance of morphological, phenological and physiological traits with respect to yield under different water regimes. One determining factor is the chlorophyll content, measured indirectly by SPAD metre. A decrease in chlorophyll under drought stress has been widely reported (Ammar et al., 2015; Siddiqui et al., 2015; Abid et al., 2017). Drought-induced early senescence leading to early chlorophyll degradation was also observed in the present study with genotypes responding differently to the stress factor. Late chlorophyll degradation in connection with prolonged photosynthetic activity is a desirable trait, especially under drought stress,

Accumulation of free proline and of carbohydrates as free sugars contributes to osmotic adjustment in plants (Yoshiba et al., 1997; Hekneby et al., 2006). An increase in free proline between 42% and 202%, under 30% of field capacity was reported in a faba bean greenhouse trial (Abid et al., 2017), These results evidenced that accumulation of proline has a role in the faba bean tolerance to drought. Our study further supports this idea, as the amount of proline was four times higher under drought stress than in controlled conditions. Siddiqui et al. (2015) reported that drought-tolerant faba bean genotypes accumulated more proline than sensitive genotypes. In contrast, Migdadi et al. (2016) found a negative correlation between proline and yield over all treatments, which was mainly due to the high accumulation of proline under drought stress conditions rather than to differences between genotypes within the treatment. This is in accordance with our study, in which there was no close correlation between proline accumulation and other traits scored in the drought stress treatment. The significant correlation in the control treatment (PY_C) could be due to limitations in the measurements, as the content of free proline and therefore the genotypic differences are very low in the absence of osmotic stress (Balko, 2005; Stoddard et al., 2006; Kabbadj et al., 2017)

Another adaptation to water stress is the accumulation of carbohydrates, one of the main components of osmoregulation in many plant species (Morgan, 1984). In the forage legume *Lotus japonicus* L., accumulation of sugars such as fructose, galactose, glucose and maltose occurred when affected by drought (Sanchez et al., 2012). In soybean leaves, about 30% of the metabolites identified were soluble sugars and sugar alcohols, which are important for plant adaptation to stresses, especially during water deficit (Benkeblia et al., 2007). These findings are supported by the present study where a 24% increase of TSS under drought conditions was observed.

Plant height in faba bean is strongly determined by the growth type. Indeterminate types reach a greater height under favorable conditions while determinate (topless) types are lower. In this study, the indeterminate types showed a broad variability in plant height. Drought stress reduced plant height and this effect was more pronounced in the tall forms. Importantly, the yield-deciding parameter is not plant height itself but the number of reproductive nodes. Drought during the reproductive phase may reduce plant height and therefore the number of reproductive nodes (Gnanasambandam et al., 2012). In our field trials, plant height in both treatments was most significantly correlated to the number of pods per plant as well as to seeds per plant.

Pods per plant, seeds per pod and seed weight are among the main yield components (Ayaz et al., 2004; Tadesse et al., 2011; Ouji et al., 2017). Under drought stress, the most susceptible developmental stage is the reproductive phase including flowering, early podding and pod setting (Muktadir et al., 2020). The early podding was the most sensitive stage reported by Mwanamwenge et al. (1999), causing a reduction in the number of pods and seeds due to high rates of abscission (Sekara et al., 2001). In our study, seeds per plant showed the closest relation to seed yield in both the control and the drought stress treatment, followed by pods per plant. Similar outcomes were reported by Pilbeam et al. (1992) and Tofiq et al. (2016). Lopez et al. (1996) reported that the number of pods per plant was most affected by drought stress during flowering, with yield reductions up to 70%.

### Yield relations and genotypes

Previous studies pointed out that high yield potential under optimal conditions does not necessarily correlate with yield under limited water irrigation (Ramirez-Vallejo and Kelly, 1998; Abdelmula et al., 1999; El-Hendawy et al., 2017). This is in agreement with the results of our study where the performance of the faba bean lines was not consistent across the two treatments. The negative correlation between yield potential (yield under non-stress) and yield stability (yield under severe stress) suggests that the use of yield stability as a target parameter for drought tolerance could lead to the selection of low yielding genotypes. Nevertheless, varieties with high yield potential will often have an advantage over varieties with lower yield potential under moderate drought stress. Whether genotypes with medium yield potential and high stress yield might have an advantage for regions with regularly occurring drought stress remains a matter of debate. For regions with occasional drought stress, the relatively close correlation between stress yield and control yield (**Figure 6**) implies that both yield under well watered and drought stress should be considered, as shown by the STI value (Fernandez, 1992). Several studies point towards STI as the most suitable index for selecting the best yielding genotypes under contrasting conditions (Fernandez, 1992; El-Hendawy et al., 2017; Memari et al., 2022). According to our results, STI exhibits a strong correlation with yield (PY) and yield components (PP and SP) both under control conditions and drought stress, and appears to be an effective index for the selection of genotypes with high yield potential under both environmental conditions.

### Correlations, cluster analysis and principal component analysis

Genotypes with higher yields in favorable as well as stress environments are the ones preferably selected in breeding programs. Nevertheless, the presence of genotype by environment (GxE) interactions is a major concern, since it may reduce correlation between genotypic and phenotypic values and slow down the selection progress (Romagosa et al., 2009).

Our results revealed a high GxE interaction for all the traits studied. Nevertheless, a clear association between the yield related traits, PP and SP and the physiological trait SPAD2 was observed through their significant correlation in both control and drought stress conditions. A moderate correlation was also found between PRO and SP in both environments. The high heritability estimates and the positive correlations of SPAD2 with PP and SP in stress conditions indicates that SPAD2 may be a suitable selection criterion for drought tolerance in faba bean.

A positive correlation between maturity and yield was found in the control condition, but not in the stress treatment. Katsoulieri et al. (2020) described that later ripening genotypes showed greater susceptibility to drought stress during early pod set compared to genotypes with early maturity. This was supported by Khan et al. (2010) as well as Manning et al. (2020), where early flowering is considered an important adaptation trait for cool-season legumes growing under semi-arid conditions.

PCA is a powerful strategy to uncover genetic factors that contribute to complex traits. It has been considered a useful complementary tool in drought tolerance screenings and in the selection of the most tolerant genotypes (Mohamamdi et al., 2017; Santos et al., 2020; Reyes et al., 2022; Weng et al., 2022). This approach has been successfully adopted in drought stress tolerance evaluations in different crop legumes such as soybean (Bouslama and Schapaugh, 1984; Toum et al., 2022), lentil (Siahsar et al., 2010), chickpea (Shah et al., 2020; Arif et al., 2021) and faba bean (Mansour et al., 2021; Afzal et al., 2022). Here we applied the same strategy to integrate multiple parameters for grouping the faba bean accessions according to their drought tolerance. Our results show that in controlled conditions, the morphological traits SP, PP and PH were the most contributing factors for the dispersion of the genotypes along the PC1 axis, while PC2 was positively loaded with the TSS in the leaf, which is often increased during grain filling and may point towards late senescence.

Under drought stress conditions, there was a shift towards traits related to a longer photosynthesis period in PC1 (as late chlorophyll content or SPAD2), resulting in a higher number of seeds per plant. On the other hand, the phenological traits EF and MAT, which are strongly related to the length of the vegetation period, were the main factors contributing to PC2. These findings were supported by investigations in *Miscanthus*, where a PCA performed with relative values and the physiological traits chlorophyll content and chlorophyll fluorescence revealed their important role in drought stress response (Weng et al., 2022). The relevance of both physiological traits for final yield under drought stress was also reported in durum wheat (Mohamamdi et al., 2017; Reyes et al., 2022), further evidencing that a delay of chlorophyll degradation during senescence can improve performance under drought conditions. These observations reinforce the idea that screening for chlorophyll content under drought conditions could significantly contribute to efficient selection in breeding programs. The PCA was also helpful to group the faba bean genotypes according to their phenotypic response (Abou-Zaitoun et al., 2018; Or-Rashid et al., 2021). It further evidences that the lines tested follow different strategies to reach high yields under drought stress.

Cluster analysis is a multivariate analysis that categorizes the genotypes into several subgroups according to their similarities, based on the capability and performance of the traits examined. This approach has been used to predict the best-performing genotypes under drought stress in other crops (Hannok et al., 2021; Mutlu et al., 2022; Aslam et al., 2023). Grouping breeding materials using multivariate data under a given condition allows plant breeders to select breeding lines more efficiently. Here we applied the same strategy to integrate phenological, physiological as well as yield components for grouping the faba bean accessions according to their drought tolerance and the clustering method was effective to differentiate the best performing genotypes under drought stress.

PCA biplot and cluster analysis demonstrate that the eight superior genotypes according to STI follow different strategies with respect to phenological, physiological and yield components. Thus, in drought conditions the genotypes of cluster 2, that included six of the best performing STI genotypes (5, 35, 64, 56, 64 and 85), were the best group in terms of performance. These genotypes are characterized not only by the highest yield (PY_DS) and STI values but also by the highest mean values for proline (PRO_DS), chlorophyll content (SPAD2_DS), plant height (PH_DS), number of pods (PP_DS), seeds per plant (SP_DS) and later maturity days (MAT_DS), thus being the most promising traits for selection. On the other hand, these genotypes displayed lower total soluble sugar content (TSS_DS) and hundred seed weight values (HSW).

In summary, our results revealed that the Stress Tolerance Index (STI) is a useful criterion for selecting drought tolerant and high yielding faba bean genotypes. Using STI and biplot analysis, eight accessions with relatively high yield under both normal and drought stress conditions were identified and six of them further validated by K-means cluster analyses. Thus, genotypes 5, 35, 64, 56, 64 and 85 could potentially be used as genetic resources in faba bean breeding programs to develop varieties with enhanced drought resistance traits. Our study also suggests that SPAD2, followed by SP and PP can be reliable indicators for selection. To bridge the gap between traditional and molecular breeding, this faba bean collection has recently been genotyped with a high-density SNP genotyping array to conduct a genome-wide association (GWAS) study for drought resistance (Gutierrez et al., 2023). After validation, the candidate genes identified can be used for marker-assisted selection to accelerate and improve faba bean yield in agricultural areas where long water deficit periods are expected.

## Data availability statement

The raw data supporting the conclusions of this article will be made available by the authors, without undue reservation.

## Author contributions

AMT and NG selected and multiplied the seeds for the experiments. CB planned and carried out the drought evaluations and processed the experimental data. CB and AT wrote the manuscript and NG contributed in the review and editing. CB and AMT acquired the financial support for the project. All authors contributed to the article and approved the submitted version.
