# Supplementary material for: Variability in drought stress response in a panel of 100 faba bean genotypes
Source: Front Plant Sci. 2023 Aug 30;14:1236147. doi: 10.3389/fpls.2023.1236147 (PMC10499557; doi:10.3389/fpls.2023.1236147)
Supplement: Supplementary file 1 [file Table_1.docx]

**Supplementary Table S1.** Composition of the test set with origin of the accessions.

| **Genotype** | **Accession ID** | **Geographical origin** | **Country** | **Supplier** |
| --- | --- | --- | --- | --- |
|  |  |  |  |  |
| 1 | **EUC_VF_003** | EUROPA | ESP | IFAPA |
| 2 | **EUC_VF_005** | EUROPA | BGR | IFAPA |
| 3 | **EUC_VF_006** | EUROPA | RUS | IFAPA |
| 4 | **EUC_VF_008** | AFRICA | EGY | IFAPA |
| 5 | **EUC_VF_009** | EUROPA | TUR | IFAPA |
| 6 | **EUC_VF_012** | ASIA | JOR | IFAPA |
| 7 | **EUC_VF_013** | ASIA | SYR | IFAPA |
| 8 | **EUC_VF_014** | ASIA | IRQ | IFAPA |
| 9 | **EUC_VF_015** | ASIA | IRQ | IFAPA |
| 10 | **EUC_VF_020** | EUROPA | TUR | IFAPA |
| 11 | **EUC_VF_021** | EUROPA | TUR | IFAPA |
| 12 | **EUC_VF_023** | AFRICA | EGY | IFAPA |
| 13 | **EUC_VF_028** | EUROPA | ESP | IFAPA |
| 14 | **EUC_VF_030** | EUROPA | ESP | IFAPA |
| 15 | **EUC_VF_044** | EUROPA | ESP | IFAPA |
| 16 | **EUC_VF_046** | EUROPA | ESP | IFAPA |
| 17 | **EUC_VF_050** | EUROPA | ESP | IFAPA |
| 18 | **EUC_VF_051** | AMERICA | PER | IFAPA |
| 19 | **EUC_VF_057** | EUROPA | ESP | IFAPA |
| 20 | **EUC_VF_058** | EUROPA | ESP | IFAPA |
| 21 | **EUC_VF_059** | EUROPA | ESP | IFAPA |
| 22 | **EUC_VF_060** | EUROPA | ESP | IFAPA |
| 23 | **EUC_VF_061** | EUROPA | ESP | IFAPA |
| 24 | **EUC_VF_062** | EUROPA | ESP | IFAPA |
| 25 | **EUC_VF_063** | EUROPA | ESP | IFAPA |
| 26 | **EUC_VF_064** | EUROPA | ESP | IFAPA |
| 27 | **EUC_VF_065** | EUROPA | ESP | IFAPA |
| 28 | **EUC_VF_067** | EUROPA | ESP | IFAPA |
| 29 | **EUC_VF_068** | EUROPA | ESP | IFAPA |
| 30 | **EUC_VF_069** | EUROPA | ESP | IFAPA |
| 31 | **EUC_VF_070** | EUROPA | ESP | IFAPA |
| 32 | **EUC_VF_071** | EUROPA | ESP | IFAPA |
| 33 | **EUC_VF_074** | EUROPA | ESP | IFAPA |
| 34 | **EUC_VF_075** | EUROPA | ESP | IFAPA |
| 35 | **EUC_VF_077** | EUROPA | RUS | IFAPA |
| 36 | **EUC_VF_079** | AFRICA | SDN | IFAPA |
| 37 | **EUC_VF_081** | ASIA | SYR | IFAPA |
| 38 | **EUC_VF_082** | ASIA | SYR | IFAPA |
| 39 | **EUC_VF_083** | ASIA | SYR | IFAPA |
| 40 | **EUC_VF_084** | ASIA | SYR | IFAPA |
| 41 | **EUC_VF_085** | UNKNOWN | 0 | IFAPA |
| 42 | **EUC_VF_091** | EUROPA | DEU | INRA |
| 43 | **EUC_VF_121** | EUROPA | AUT | INRA |
| 44 | **EUC_VF_124** | ASIA | CHN | INRA |
| 45 | **EUC_VF_125** | ASIA | CHN | INRA |
| 46 | **EUC_VF_127** | ASIA | CHN | INRA |
| 47 | **EUC_VF_128** | ASIA | CHN | INRA |
| 48 | **EUC_VF_129** | EUROPA | FRA | INRA |
| 49 | **EUC_VF_130** | EUROPA | DEU | INRA |
| 50 | **EUC_VF_132** | AMERICA | CAN | INRA |
| 51 | **EUC_VF_137** | EUROPA | GBR | INRA |
| 52 | **EUC_VF_169** | AFRICA | TUN | IFAPA |
| 53 | **EUC_VF_170** | EUROPA | ESP | IFAPA |
| 54 | **EUC_VF_171** | EUROPA | GBR | IFAPA |
| 55 | **EUC_VF_173** | EUROPA | RUS | IFAPA |
| 56 | **EUC_VF_174** | ASIA | IRN | IFAPA |
| 57 | **EUC_VF_185** | EUROPA | CZE | INRA |
| 58 | **EUC_VF_187** | EUROPA | FRA | INRA |
| 59 | **EUC_VF_188** | EUROPA | FRA | INRA |
| 60 | **EUC_VF_189** | AFRICA | ETH | INRA |
| 61 | **EUC_VF_190** | ASIA | JPN | INRA |
| 62 | **EUC_VF_191** | ASIA | IRN | INRA |
| 63 | **EUC_VF_192** | ASIA | NPL | INRA |
| 64 | **EUC_VF_194** | AFRICA | EGY | INRA |
| 65 | **EUC_VF_293** | ASIA | CHN | ICARDA |
| 66 | **EUC_VF_294** | ASIA | CHN | ICARDA |
| 67 | **EUC_VF_295** | ASIA | CHN | ICARDA |
| 68 | **EUC_VF_296** | ASIA | CHN | ICARDA |
| 69 | **EUC_VF_297** | ASIA | CHN | ICARDA |
| 70 | **EUC_VF_298** | ASIA | CHN | ICARDA |
| 71 | **EUC_VF_299** | ASIA | CHN | ICARDA |
| 72 | **EUC_VF_300** | ASIA | CHN | ICARDA |
| 73 | **EUC_VF_301** | ASIA | CHN | ICARDA |
| 74 | **EUC_VF_302** | ASIA | CHN | ICARDA |
| 75 | **EUC_VF_303** | ASIA | CHN | ICARDA |
| 76 | **EUC_VF_304** | UNKNOWN | - | ICARDA |
| 77 | **EUC_VF_305** | UNKNOWN | - | ICARDA |
| 78 | **EUC_VF_306** | UNKNOWN | - | ICARDA |
| 79 | **EUC_VF_307** | UNKNOWN | - | ICARDA |
| 80 | **EUC_VF_330** | UNKNOWN | - | ICARDA |
| 81 | **EUC_VF_331** | UNKNOWN | - | ICARDA |
| 82 | **EUC_VF_333** | UNKNOWN | - | ICARDA |
| 83 | **EUC_VF_334** | UNKNOWN | - | ICARDA |
| 84 | **EUC_VF_335** | UNKNOWN | - | ICARDA |
| 85 | **EUC_VF_336** | AFRICA | EGY | ICARDA |
| 86 | **EUC_VF_337** | AFRICA | EGY | ICARDA |
| 87 | **EUC_VF_338** | UNKNOWN | - | ICARDA |
| 88 | **EUC_VF_339** | UNKNOWN | - | ICARDA |
| 89 | **EUC_VF_340** | UNKNOWN | - | ICARDA |
| 90 | **EUC_VF_342** | UNKNOWN | - | ICARDA |
| 91 | **EUC_VF_343** | UNKNOWN | - | ICARDA |
| 92 | **EUC_VF_344** | UNKNOWN | - | ICARDA |
| 93 | **EUC_VF_345** | UNKNOWN | - | ICARDA |
| 94 | **EUC_VF_346** | UNKNOWN | - | ICARDA |
| 95 | **EUC_VF_347** | UNKNOWN | - | ICARDA |
| 96 | **EUC_VF_348** | UNKNOWN | - | ICARDA |
| 97 | **EUC_VF_349** | UNKNOWN | - | ICARDA |
| 98 | **EUC_VF_350** | UNKNOWN | - | ICARDA |
| 99 | **EUC_VF_351** | UNKNOWN | - | ICARDA |
| 100 | **EUC_VF_352** | UNKNOWN | - | ICARDA |
