# Supplementary material for: Variability in drought stress response in a panel of 100 faba bean genotypes
Source: Front Plant Sci. 2023 Aug 30;14:1236147. doi: 10.3389/fpls.2023.1236147 (PMC10499557; doi:10.3389/fpls.2023.1236147)
Supplement: Supplementary file 2 [file Table_2.docx]

**Supplementary Table S2.** Description and data of the field management activities.

| **Activity** | **Description** | **Year 2019** | **Year 2020** |
| --- | --- | --- | --- |
| **Sowing** | Single row seed drill | 02 April | 06 April |
| **Herbicide treatment** | Single application of STOMP AQUA (BASF)  3 l / ha | Pre-emergence (05 April) | Pre-emergence (08 April) |
| **Fertilization treatment** | Fertilization with phosphorus (P) and potassium (K) after standard soil test analyses. | The supply level was high (P) to sufficient (K). No nitrogen (N) fertilizer was applied. | The supply level was high (P) to sufficient (K). No (N) fertilizer was applied. |
| **Emergence** | Time of plant emergence | 20 to 25 April | 20 to 26 April |
| **Insecticide treatment** | KAISO SORBIE (Nufarm)  150 g/ ha to control spider mites. | 07 May | 08 May |
| **Weeding** | Manually | After emergence of weeds | After emergence of weeds |
| **Begin flowering (BF)** | BBCH 60 (First flowers open) | 30 May to 16 June | 02 May to 19 June |
| **Irrigation** | Drip irrigation | See materials and methods | See materials and methods |
| **Drought treatment** | Complete restriction of water  (Stop of irrigation, shelter cover plots in case of rainfall) | 05 June | 04 June |
| **SPAD1** | Indirect measurement of the chlorophyll content at the beginning of stress treatment | 13 June | 09 June |
| **Insecticide treatment** | KARATE ZEON (Syngenta) 0.75 ml/ha to control spider mites. | No treatment | 10 June |
| **Sampling**  **PRO and TSS** | Leaf sampling for determination of proline content and total soluble sugars content | 27 June | 07 July |
| **SPAD2** | Indirect measurement of the chlorophyll content after 4 weeks stress development | 01 July | 08 July |
| **Maturity date (MAT)** | BBCH 89 (Nearly all pods dark, seeds dry and hard) | Shelter: 12 July to 12 August  Field: 26 July to 28 August | Shelter: 20 July to 17 August  Field: 28 July to 28 August |
