# Supplementary material for: Variability in drought stress response in a panel of 100 faba bean genotypes
Source: Front Plant Sci. 2023 Aug 30;14:1236147. doi: 10.3389/fpls.2023.1236147 (PMC10499557; doi:10.3389/fpls.2023.1236147)
Supplement: Supplementary file 3 [file Table_3.docx]

**Supplementary Table S3.** LS Means of all traits described in table 2 for the 100 genotypes.

| **Genotype** | **TSS C** | **TSS DS** | **PRO C** | **PRO DS** | **SPAD2 C** | **SPAD2 DS** | **DiffSPAD C** | **DiffSPAD DS** | **EF C** | **EF DS** | **MAT C** | **MAT DS** | **PH C** | **PH DS** | **PP C** | **PP DS** | **SP C** | **SP DS** | **HSW C** | **HSW DS** | **PY C** | **PY DS** | **STI** |
| --- | --- | --- | --- | --- | --- | --- | --- | --- | --- | --- | --- | --- | --- | --- | --- | --- | --- | --- | --- | --- | --- | --- | --- |
| **1** | 906,46 | 1053,59 | 2,40 | 4,12 | 45,5 | 19,5 | -8,2 | 20,5 | 87 | 84 | 131 | 115 | 58,7 | 54,6 | 10,5 | 6,6 | 21,3 | 14,2 | 64,81 | 59,56 | 0,139 | 0,086 | 0,50 |
| **2** | 1002,12 | 1119,59 | 2,79 | 5,22 | 46,1 | 28,3 | -7,5 | 12,3 | 99 | 91 | 132 | 117 | 77,2 | 63,8 | 14,1 | 9,5 | 27,2 | 17,3 | 69,77 | 65,49 | 0,188 | 0,115 | 0,90 |
| **3** | 1021,04 | 1266,05 | 2,50 | 13,31 | 51,5 | 22,8 | -9,8 | 19,8 | 88 | 83 | 128 | 115 | 58,0 | 55,5 | 11,0 | 7,5 | 21,6 | 12,8 | 84,32 | 78,69 | 0,178 | 0,100 | 0,74 |
| **4** | 982,62 | 1073,61 | 2,23 | 3,90 | 37,2 | 20,6 | -0,6 | 17,8 | 91 | 83 | 123 | 108 | 53,0 | 53,7 | 8,9 | 6,6 | 21,7 | 17,3 | 51,02 | 40,60 | 0,100 | 0,071 | 0,29 |
| **5** | 1153,01 | 1966,26 | 2,73 | 56,39 | 49,1 | 30,7 | -10,3 | 6,8 | 93 | 86 | 131 | 117 | 69,7 | 58,8 | 13,5 | 7,1 | 34,0 | 15,7 | 77,09 | 67,64 | 0,266 | 0,106 | 1,18 |
| **6** | 950,52 | 1133,61 | 3,15 | 4,68 | 39,4 | 21,9 | -2,2 | 16,5 | 88 | 83 | 124 | 110 | 59,2 | 50,6 | 10,5 | 5,7 | 26,7 | 13,6 | 65,84 | 57,51 | 0,175 | 0,078 | 0,57 |
| **7** | 900,99 | 1074,48 | 2,28 | 2,62 | 41,7 | 20,5 | -5,0 | 16,3 | 89 | 82 | 124 | 110 | 58,7 | 52,5 | 10,4 | 6,1 | 23,7 | 12,5 | 86,21 | 72,34 | 0,205 | 0,091 | 0,78 |
| **8** | 1027,49 | 1571,36 | 2,23 | 2,94 | 47,2 | 22,7 | -6,6 | 18,5 | 94 | 87 | 129 | 115 | 60,1 | 55,4 | 8,6 | 5,2 | 21,4 | 13,4 | 67,34 | 56,01 | 0,146 | 0,069 | 0,42 |
| **9** | 1121,74 | 1569,28 | 2,23 | 11,87 | 46,9 | 29,3 | -8,7 | 11,9 | 103 | 96 | 133 | 118 | 61,4 | 56,2 | 13,4 | 7,0 | 30,0 | 15,9 | 63,15 | 50,99 | 0,167 | 0,072 | 0,50 |
| **10** | 1075,81 | 1453,05 | 2,11 | 6,95 | 48,8 | 23,6 | -8,1 | 19,4 | 101 | 93 | 131 | 117 | 55,2 | 52,2 | 8,3 | 5,6 | 18,4 | 12,6 | 96,72 | 71,67 | 0,182 | 0,091 | 0,69 |
| **11** | 802,98 | 1233,68 | 2,74 | 13,48 | 40,3 | 24,3 | -5,5 | 13,5 | 98 | 84 | 130 | 117 | 67,2 | 53,7 | 9,5 | 5,5 | 24,5 | 12,6 | 85,37 | 72,73 | 0,208 | 0,090 | 0,78 |
| **12** | 1036,63 | 1349,62 | 2,10 | 11,68 | 38,3 | 24,8 | 0,2 | 15,5 | 96 | 84 | 124 | 112 | 49,1 | 49,7 | 9,0 | 6,5 | 20,0 | 14,9 | 64,96 | 57,74 | 0,130 | 0,086 | 0,47 |
| **13** | 1034,57 | 1409,20 | 2,15 | 5,36 | 47,0 | 28,6 | -7,7 | 9,3 | 96 | 85 | 129 | 115 | 56,5 | 53,4 | 9,9 | 6,3 | 23,1 | 13,1 | 73,52 | 68,59 | 0,167 | 0,091 | 0,63 |
| **14** | 1074,47 | 1647,23 | 1,85 | 7,13 | 46,7 | 21,2 | -6,0 | 21,7 | 102 | 91 | 133 | 116 | 65,9 | 59,3 | 7,2 | 4,1 | 17,3 | 9,0 | 96,46 | 79,89 | 0,169 | 0,072 | 0,51 |
| **15** | 911,23 | 1343,62 | 2,47 | 12,03 | 57,4 | 30,9 | -15,1 | 12,8 | 95 | 84 | 130 | 114 | 69,6 | 56,9 | 15,2 | 9,1 | 29,4 | 16,4 | 57,29 | 44,52 | 0,164 | 0,075 | 0,51 |
| **16** | 1053,20 | 1110,34 | 2,12 | 5,23 | 49,5 | 21,6 | -8,5 | 21,3 | 94 | 85 | 126 | 114 | 71,9 | 57,0 | 10,5 | 5,0 | 24,3 | 11,5 | 69,89 | 60,94 | 0,169 | 0,070 | 0,49 |
| **17** | 1024,02 | 1626,54 | 2,23 | 13,52 | 44,7 | 27,4 | -10,1 | 9,3 | 107 | 90 | 131 | 121 | 67,5 | 55,5 | 9,5 | 5,9 | 20,3 | 13,5 | 63,03 | 61,34 | 0,118 | 0,083 | 0,41 |
| **18** | 880,26 | 998,16 | 2,07 | 3,19 | 40,0 | 19,8 | -7,6 | 14,3 | 99 | 87 | 125 | 113 | 70,2 | 61,2 | 12,4 | 7,5 | 21,5 | 12,8 | 73,88 | 60,94 | 0,159 | 0,078 | 0,52 |
| **19** | 1147,23 | 1237,53 | 3,14 | 4,72 | 48,5 | 24,8 | -10,1 | 16,5 | 99 | 85 | 127 | 110 | 61,6 | 55,2 | 11,3 | 6,4 | 25,5 | 15,0 | 49,87 | 45,32 | 0,123 | 0,069 | 0,35 |
| **20** | 1196,59 | 1549,58 | 2,00 | 5,21 | 49,0 | 33,3 | -8,3 | 10,3 | 104 | 87 | 132 | 117 | 78,5 | 67,1 | 16,9 | 8,9 | 39,1 | 20,3 | 37,58 | 34,36 | 0,148 | 0,071 | 0,44 |
| **21** | 1286,78 | 1477,69 | 2,36 | 19,94 | 47,0 | 24,3 | -9,3 | 15,3 | 102 | 84 | 132 | 119 | 57,5 | 53,0 | 12,9 | 5,6 | 22,0 | 13,0 | 73,40 | 66,91 | 0,162 | 0,087 | 0,58 |
| **22** | 1195,74 | 1499,41 | 1,79 | 2,73 | 37,2 | 16,2 | 0,4 | 24,0 | 105 | 89 | 129 | 116 | 53,9 | 45,2 | 6,3 | 3,5 | 10,5 | 5,8 | 56,43 | 51,48 | 0,059 | 0,031 | 0,08 |
| **23** | 1390,43 | 1780,04 | 1,92 | 2,86 | 49,0 | 20,0 | -6,9 | 24,8 | 112 | 92 | 133 | 124 | 58,4 | 52,7 | 12,5 | 5,9 | 18,7 | 9,1 | 62,46 | 49,14 | 0,113 | 0,044 | 0,21 |
| **24** | 1066,35 | 1456,15 | 1,83 | 3,06 | 40,5 | 19,6 | -7,7 | 15,2 | 97 | 95 | 126 | 120 | 63,3 | 61,1 | 6,0 | 3,7 | 15,1 | 8,4 | 94,83 | 103,24 | 0,140 | 0,085 | 0,50 |
| **25** | 1112,82 | 1532,83 | 2,39 | 5,90 | 43,7 | 27,9 | -2,9 | 16,0 | 96 | 94 | 128 | 123 | 61,4 | 56,9 | 9,6 | 5,4 | 17,9 | 10,2 | 60,04 | 75,44 | 0,110 | 0,077 | 0,35 |
| **26** | 770,94 | 1043,26 | 2,04 | 8,36 | 49,2 | 26,6 | -11,9 | 12,8 | 98 | 89 | 128 | 116 | 61,5 | 51,5 | 7,5 | 4,7 | 19,7 | 11,4 | 115,37 | 91,09 | 0,228 | 0,104 | 0,99 |
| **27** | 1217,08 | 1583,78 | 2,04 | 1,79 | 42,6 | 21,3 | -8,9 | 15,7 | 103 | 84 | 130 | 118 | 63,6 | 56,8 | 10,1 | 5,7 | 24,6 | 13,2 | 57,21 | 47,54 | 0,147 | 0,064 | 0,39 |
| **28** | 877,36 | 1430,85 | 1,99 | 11,35 | 51,3 | 31,5 | -10,9 | 9,1 | 101 | 84 | 129 | 119 | 53,9 | 49,9 | 5,5 | 3,6 | 15,3 | 8,6 | 96,02 | 91,16 | 0,147 | 0,080 | 0,49 |
| **29** | 942,18 | 1071,37 | 1,95 | 5,30 | 45,3 | 25,0 | -4,9 | 19,9 | 92 | 83 | 123 | 113 | 62,5 | 55,8 | 9,0 | 6,2 | 24,2 | 14,5 | 74,17 | 67,17 | 0,168 | 0,100 | 0,70 |
| **30** | 1125,51 | 1358,34 | 2,09 | 9,62 | 43,8 | 19,6 | -4,6 | 22,2 | 93 | 90 | 125 | 112 | 66,6 | 61,6 | 13,4 | 8,1 | 28,4 | 17,0 | 70,85 | 56,39 | 0,199 | 0,095 | 0,79 |
| **31** | 1239,27 | 1638,70 | 1,63 | 4,40 | 40,4 | 18,1 | 0,0 | 25,2 | 102 | 83 | 126 | 113 | 59,6 | 59,9 | 6,7 | 4,8 | 15,6 | 12,4 | 85,33 | 71,77 | 0,129 | 0,089 | 0,48 |
| **32** | 999,76 | 1304,12 | 2,20 | 7,51 | 47,3 | 21,4 | -9,1 | 19,8 | 100 | 86 | 126 | 118 | 57,9 | 50,7 | 12,6 | 8,0 | 20,0 | 12,4 | 72,36 | 65,28 | 0,141 | 0,083 | 0,49 |
| **33** | 1114,39 | 1032,04 | 1,91 | 4,05 | 48,6 | 23,7 | -8,6 | 18,5 | 98 | 84 | 128 | 115 | 68,1 | 58,5 | 10,4 | 5,7 | 25,0 | 14,6 | 86,20 | 68,25 | 0,206 | 0,099 | 0,85 |
| **34** | 1350,38 | 1506,20 | 2,66 | 6,35 | 34,5 | 27,2 | 3,9 | 14,2 | 90 | 83 | 123 | 114 | 66,6 | 58,8 | 12,5 | 7,6 | 29,1 | 17,0 | 49,39 | 49,36 | 0,148 | 0,086 | 0,53 |
| **35** | 1223,94 | 1338,55 | 2,37 | 7,36 | 48,7 | 32,5 | -8,5 | 7,6 | 97 | 85 | 130 | 116 | 86,9 | 68,1 | 19,2 | 9,5 | 47,1 | 20,6 | 62,37 | 54,79 | 0,289 | 0,114 | 1,37 |
| **36** | 931,05 | 1368,83 | 2,01 | 7,91 | 37,1 | 14,5 | -2,8 | 23,4 | 100 | 83 | 130 | 110 | 55,4 | 52,8 | 8,0 | 5,3 | 22,2 | 14,0 | 78,32 | 62,86 | 0,167 | 0,088 | 0,61 |
| **37** | 935,40 | 1075,39 | 2,35 | 3,71 | 44,3 | 20,4 | -6,1 | 19,8 | 95 | 83 | 124 | 110 | 56,2 | 56,1 | 12,2 | 8,0 | 33,1 | 20,2 | 59,46 | 51,51 | 0,186 | 0,105 | 0,81 |
| **38** | 1316,23 | 1497,83 | 2,21 | 7,05 | 48,0 | 25,6 | -6,2 | 16,7 | 96 | 86 | 125 | 116 | 56,4 | 51,6 | 8,9 | 5,3 | 19,7 | 11,7 | 84,85 | 69,65 | 0,166 | 0,083 | 0,57 |
| **39** | 961,87 | 1231,19 | 2,09 | 6,71 | 41,2 | 26,9 | -6,0 | 8,4 | 96 | 86 | 128 | 114 | 64,5 | 60,1 | 10,4 | 7,5 | 23,2 | 15,7 | 70,97 | 64,60 | 0,162 | 0,102 | 0,69 |
| **40** | 980,13 | 1201,53 | 2,46 | 2,12 | 40,9 | 22,3 | -1,3 | 14,3 | 101 | 90 | 127 | 117 | 42,1 | 37,1 | 4,5 | 3,7 | 12,6 | 10,1 | 48,99 | 59,31 | 0,062 | 0,062 | 0,16 |
| **41** | 922,96 | 1156,65 | 2,09 | 6,23 | 45,9 | 19,4 | -7,7 | 20,5 | 86 | 83 | 120 | 111 | 63,2 | 58,8 | 11,9 | 7,9 | 32,1 | 20,4 | 56,68 | 52,68 | 0,186 | 0,107 | 0,83 |
| **42** | 1211,29 | 1208,07 | 4,73 | 24,28 | 49,1 | 48,3 | -6,4 | -4,9 | 104 | 89 | 132 | 124 | 93,8 | 69,9 | 20,0 | 8,6 | 56,8 | 23,1 | 39,12 | 40,12 | 0,227 | 0,095 | 0,90 |
| **43** | 1249,57 | 1154,44 | 3,77 | 18,10 | 47,9 | 47,1 | -4,7 | -2,7 | 100 | 90 | 134 | 119 | 102,3 | 71,3 | 21,5 | 8,9 | 56,3 | 19,5 | 36,79 | 37,27 | 0,215 | 0,072 | 0,64 |
| **44** | 1170,14 | 1517,22 | 1,75 | 2,03 | 35,6 | 32,5 | 0,7 | 1,9 | 90 | 84 | 118 | 113 | 48,9 | 45,4 | 6,0 | 4,8 | 9,6 | 7,8 | 47,86 | 52,70 | 0,047 | 0,042 | 0,08 |
| **45** | 1213,66 | 1553,44 | 3,18 | 6,79 | 36,9 | 23,1 | -0,7 | 13,0 | 95 | 87 | 126 | 118 | 72,1 | 64,4 | 13,7 | 7,6 | 23,3 | 12,5 | 55,55 | 53,85 | 0,128 | 0,068 | 0,36 |
| **46** | 1383,77 | 2242,39 | 1,86 | 3,44 | 32,9 | 11,5 | 1,5 | 24,0 | 102 | 91 | 123 | 115 | 50,4 | 50,2 | 5,4 | 5,0 | 8,9 | 8,4 | 60,82 | 57,06 | 0,053 | 0,048 | 0,11 |
| **47** | 1214,65 | 1585,70 | 2,13 | 2,94 | 31,1 | 13,3 | 2,2 | 21,5 | 97 | 83 | 121 | 111 | 45,2 | 48,5 | 8,6 | 7,4 | 12,1 | 10,2 | 61,43 | 53,66 | 0,075 | 0,055 | 0,17 |
| **48** | 1434,48 | 1265,68 | 2,38 | 8,68 | 42,5 | 34,5 | -7,2 | 0,3 | 100 | 87 | 134 | 118 | 84,2 | 68,9 | 19,2 | 10,0 | 49,7 | 21,0 | 41,19 | 38,73 | 0,205 | 0,081 | 0,69 |
| **49** | 1054,73 | 1219,42 | 4,07 | 33,81 | 50,5 | 44,5 | -9,0 | -4,0 | 104 | 85 | 133 | 120 | 93,0 | 66,4 | 27,1 | 9,8 | 77,9 | 25,2 | 42,66 | 40,15 | 0,321 | 0,100 | 1,33 |
| **50** | 1273,16 | 1333,30 | 1,99 | 15,25 | 40,6 | 34,1 | -4,8 | 2,6 | 102 | 89 | 130 | 114 | 83,1 | 58,1 | 22,2 | 9,8 | 59,3 | 27,0 | 30,27 | 26,07 | 0,180 | 0,072 | 0,54 |
| **51*** |  |  |  |  |  |  |  |  |  |  |  |  |  |  |  |  |  |  |  |  |  |  |  |
| **52** | 1048,26 | 1322,01 | 1,97 | 9,58 | 48,8 | 24,1 | -6,4 | 20,5 | 93 | 83 | 129 | 114 | 61,3 | 54,6 | 10,9 | 6,8 | 26,5 | 15,8 | 77,43 | 62,30 | 0,212 | 0,099 | 0,88 |
| **53** | 980,77 | 1496,11 | 2,24 | 5,99 | 47,3 | 17,9 | -6,6 | 22,3 | 86 | 82 | 120 | 113 | 66,4 | 55,1 | 14,2 | 7,4 | 38,1 | 19,1 | 48,65 | 46,01 | 0,188 | 0,089 | 0,70 |
| **54** | 991,20 | 1246,18 | 2,30 | 4,46 | 53,4 | 26,1 | -12,6 | 16,6 | 99 | 84 | 126 | 114 | 63,9 | 51,7 | 13,1 | 7,6 | 31,4 | 17,9 | 73,84 | 54,53 | 0,231 | 0,097 | 0,93 |
| **55** | 905,59 | 1422,07 | 2,21 | 5,35 | 43,8 | 23,2 | -6,7 | 15,4 | 88 | 88 | 122 | 116 | 60,8 | 50,3 | 11,3 | 7,9 | 28,8 | 18,4 | 51,79 | 44,77 | 0,146 | 0,084 | 0,51 |
| **56** | 1289,77 | 1346,34 | 2,11 | 5,59 | 41,3 | 30,0 | -6,4 | 5,7 | 110 | 90 | 132 | 123 | 84,0 | 71,6 | 21,8 | 12,0 | 38,5 | 19,3 | 61,97 | 52,01 | 0,235 | 0,103 | 1,00 |
| **57** | 1229,19 | 1483,59 | 2,11 | 14,51 | 45,8 | 41,9 | -3,5 | -0,8 | 107 | 87 | 134 | 121 | 87,9 | 55,9 | 12,1 | 5,1 | 28,9 | 11,8 | 49,59 | 56,50 | 0,146 | 0,067 | 0,41 |
| **58** | 1002,79 | 1364,97 | 2,16 | 7,28 | 41,9 | 24,9 | -7,9 | 11,7 | 99 | 85 | 129 | 115 | 52,5 | 51,1 | 6,8 | 5,1 | 14,6 | 10,2 | 104,29 | 91,99 | 0,143 | 0,095 | 0,56 |
| **59** | 1146,37 | 1195,71 | 4,56 | 21,38 | 48,3 | 51,3 | -7,7 | -6,9 | 119 | 100 | 139 | 125 | 87,8 | 65,8 | 13,2 | 8,4 | 33,6 | 21,6 | 32,05 | 29,31 | 0,114 | 0,066 | 0,31 |
| **60** | 1094,34 | 1344,01 | 1,77 | 3,49 | 38,2 | 21,9 | -4,2 | 13,1 | 96 | 88 | 122 | 114 | 59,2 | 48,3 | 10,2 | 7,9 | 27,2 | 20,5 | 38,59 | 38,10 | 0,102 | 0,080 | 0,34 |
| **61** | 1070,84 | 1290,80 | 2,43 | 5,86 | 32,1 | 26,3 | -1,2 | 6,7 | 99 | 84 | 123 | 113 | 60,2 | 56,6 | 8,3 | 4,7 | 21,9 | 11,9 | 54,68 | 55,89 | 0,115 | 0,066 | 0,32 |
| **62** | 1013,64 | 1328,97 | 1,99 | 3,04 | 41,5 | 20,6 | -3,2 | 19,6 | 94 | 83 | 120 | 109 | 58,9 | 56,3 | 10,9 | 8,8 | 22,7 | 19,8 | 41,76 | 38,96 | 0,092 | 0,077 | 0,29 |
| **63** | 982,79 | 1009,04 | 2,36 | 4,43 | 35,8 | 14,5 | -5,2 | 18,4 | 91 | 83 | 122 | 106 | 52,4 | 43,2 | 11,9 | 6,9 | 28,0 | 16,2 | 61,20 | 49,12 | 0,168 | 0,080 | 0,56 |
| **64** | 1459,92 | 1235,05 | 3,18 | 12,26 | 43,4 | 34,9 | 1,7 | 4,3 | 98 | 84 | 136 | 117 | 97,1 | 65,5 | 20,7 | 7,7 | 58,2 | 19,3 | 52,08 | 52,61 | 0,301 | 0,105 | 1,32 |
| **65** | 1137,65 | 1608,02 | 2,49 | 16,01 | 42,2 | 20,1 | -3,1 | 22,1 | 102 | 88 | 125 | 113 | 69,4 | 59,6 | 7,7 | 4,7 | 16,6 | 10,4 | 101,69 | 88,03 | 0,168 | 0,093 | 0,65 |
| **66** | 1273,91 | 1568,38 | 2,38 | 5,47 | 29,2 | 11,5 | 5,2 | 24,3 | 100 | 87 | 124 | 114 | 65,7 | 63,1 | 12,5 | 9,1 | 22,9 | 17,4 | 40,54 | 42,71 | 0,088 | 0,068 | 0,25 |
| **67** | 1388,15 | 2040,75 | 1,78 | 4,28 | 36,1 | 14,1 | -4,0 | 19,1 | 97 | 91 | 126 | 118 | 65,1 | 58,2 | 6,7 | 4,4 | 12,4 | 8,5 | 85,75 | 78,40 | 0,105 | 0,065 | 0,28 |
| **68** | 1320,51 | 1537,76 | 2,04 | 4,13 | 34,5 | 18,6 | -1,6 | 17,0 | 97 | 93 | 128 | 117 | 67,5 | 54,5 | 8,1 | 4,2 | 14,5 | 7,9 | 72,64 | 62,35 | 0,103 | 0,051 | 0,22 |
| **69** | 1217,54 | 1665,07 | 1,68 | 3,66 | 41,7 | 19,5 | -5,6 | 19,3 | 101 | 90 | 129 | 117 | 67,8 | 61,5 | 8,6 | 6,1 | 14,1 | 9,5 | 71,19 | 62,61 | 0,109 | 0,063 | 0,28 |
| **70** | 1358,88 | 1675,06 | 1,75 | 13,77 | 33,0 | 19,4 | 4,5 | 18,7 | 101 | 94 | 129 | 120 | 63,6 | 61,6 | 7,1 | 5,1 | 12,4 | 7,8 | 69,72 | 61,32 | 0,092 | 0,045 | 0,17 |
| **71** | 1239,45 | 1494,35 | 1,98 | 3,67 | 34,0 | 26,6 | 0,0 | 10,3 | 108 | 93 | 132 | 125 | 68,4 | 62,7 | 11,3 | 6,5 | 18,9 | 10,7 | 66,57 | 62,75 | 0,126 | 0,067 | 0,35 |
| **72** | 1146,63 | 1198,89 | 1,99 | 3,48 | 46,0 | 22,2 | -3,9 | 20,8 | 101 | 93 | 129 | 115 | 63,4 | 57,7 | 9,6 | 5,7 | 16,9 | 9,8 | 80,12 | 68,30 | 0,136 | 0,067 | 0,38 |
| **73** | 1709,43 | 1970,47 | 1,63 | 6,59 | 34,0 | 15,5 | 3,9 | 23,6 | 108 | 89 | 131 | 115 | 65,2 | 60,2 | 7,5 | 4,1 | 11,4 | 6,6 | 80,26 | 74,41 | 0,089 | 0,049 | 0,18 |
| **74** | 1494,86 | 1776,97 | 1,55 | 2,17 | 36,7 | 18,4 | -0,9 | 19,4 | 95 | 86 | 125 | 115 | 65,2 | 62,6 | 8,0 | 5,1 | 16,3 | 10,1 | 56,03 | 53,87 | 0,090 | 0,055 | 0,21 |
| **75** | 983,91 | 1238,03 | 2,79 | 8,26 | 42,9 | 26,0 | -6,5 | 11,5 | 97 | 85 | 131 | 116 | 81,4 | 66,5 | 13,0 | 5,5 | 29,9 | 11,7 | 89,68 | 84,29 | 0,271 | 0,099 | 1,12 |
| **76** | 1157,99 | 1200,09 | 2,27 | 7,74 | 36,4 | 19,5 | 0,2 | 20,5 | 91 | 84 | 128 | 113 | 58,2 | 54,9 | 6,7 | 4,5 | 20,5 | 13,0 | 67,92 | 63,98 | 0,133 | 0,083 | 0,46 |
| **77** | 1078,99 | 1392,58 | 2,32 | 12,72 | 48,0 | 19,8 | -5,5 | 22,8 | 93 | 83 | 132 | 115 | 67,3 | 60,4 | 7,4 | 4,5 | 21,2 | 13,5 | 86,76 | 76,65 | 0,180 | 0,104 | 0,78 |
| **78** | 1176,91 | 1393,60 | 2,02 | 8,79 | 39,2 | 17,5 | -3,3 | 23,1 | 88 | 83 | 125 | 113 | 55,5 | 51,4 | 7,4 | 4,7 | 20,1 | 12,7 | 73,49 | 66,22 | 0,145 | 0,085 | 0,52 |
| **79** | 1134,03 | 1233,97 | 2,04 | 3,17 | 44,2 | 22,7 | -5,2 | 19,5 | 99 | 85 | 127 | 113 | 56,1 | 48,2 | 7,8 | 4,8 | 22,1 | 13,0 | 84,25 | 70,29 | 0,184 | 0,092 | 0,71 |
| **80** | 1091,31 | 1033,07 | 2,16 | 6,83 | 41,2 | 17,7 | -6,3 | 18,4 | 89 | 83 | 123 | 110 | 57,8 | 52,1 | 10,1 | 6,0 | 24,1 | 14,5 | 68,58 | 61,21 | 0,151 | 0,090 | 0,56 |
| **81** | 945,97 | 1089,45 | 2,15 | 3,52 | 44,1 | 20,9 | -4,7 | 19,5 | 87 | 83 | 121 | 110 | 51,9 | 51,1 | 7,5 | 5,7 | 19,7 | 15,2 | 72,54 | 62,37 | 0,144 | 0,094 | 0,57 |
| **82** | 890,93 | 1458,20 | 2,51 | 14,06 | 39,7 | 17,7 | -6,7 | 17,9 | 102 | 82 | 130 | 110 | 62,1 | 55,1 | 8,8 | 6,7 | 18,7 | 15,5 | 68,79 | 59,27 | 0,128 | 0,094 | 0,50 |
| **83** | 976,71 | 1527,22 | 2,48 | 8,99 | 38,2 | 18,9 | -4,8 | 15,3 | 89 | 84 | 123 | 111 | 59,8 | 55,3 | 7,4 | 5,2 | 18,1 | 11,7 | 63,94 | 56,15 | 0,109 | 0,066 | 0,30 |
| **84** | 956,35 | 1095,02 | 1,87 | 2,82 | 45,6 | 18,2 | -8,3 | 21,4 | 95 | 84 | 125 | 113 | 55,9 | 54,2 | 9,5 | 6,1 | 21,0 | 14,6 | 60,77 | 60,22 | 0,128 | 0,089 | 0,47 |
| **85** | 1153,47 | 1102,49 | 2,43 | 12,64 | 39,0 | 34,3 | -6,1 | -0,5 | 110 | 88 | 136 | 121 | 86,9 | 60,9 | 20,2 | 8,7 | 46,4 | 18,0 | 56,34 | 55,89 | 0,252 | 0,102 | 1,07 |
| **86** | 1076,94 | 1398,25 | 1,97 | 5,91 | 40,5 | 22,5 | -5,2 | 15,3 | 89 | 83 | 126 | 116 | 56,8 | 50,3 | 10,2 | 5,8 | 20,4 | 12,6 | 79,69 | 80,98 | 0,161 | 0,106 | 0,71 |
| **87** | 1114,75 | 1344,19 | 1,86 | 2,96 | 36,9 | 16,6 | -2,9 | 18,6 | 92 | 83 | 123 | 111 | 54,4 | 51,1 | 7,4 | 5,1 | 19,4 | 11,3 | 83,84 | 74,57 | 0,158 | 0,087 | 0,57 |
| **88** | 1127,37 | 1333,33 | 1,77 | 4,64 | 45,5 | 23,4 | -2,3 | 21,8 | 97 | 84 | 130 | 115 | 56,2 | 56,8 | 10,6 | 7,1 | 20,4 | 15,5 | 71,40 | 65,77 | 0,143 | 0,104 | 0,62 |
| **89** | 931,92 | 1114,42 | 1,67 | 5,38 | 40,1 | 20,7 | -2,3 | 19,5 | 91 | 83 | 124 | 112 | 46,2 | 45,2 | 7,9 | 5,8 | 19,5 | 14,5 | 64,13 | 62,57 | 0,123 | 0,093 | 0,48 |
| **90** | 1000,34 | 1023,04 | 2,07 | 5,12 | 44,2 | 22,5 | -5,3 | 18,8 | 94 | 86 | 124 | 113 | 60,4 | 53,9 | 8,5 | 4,9 | 23,9 | 13,6 | 73,34 | 59,97 | 0,164 | 0,082 | 0,56 |
| **91** | 1138,56 | 1440,24 | 2,14 | 8,32 | 41,4 | 17,6 | -5,1 | 20,3 | 95 | 82 | 126 | 112 | 64,7 | 57,2 | 8,6 | 5,6 | 22,0 | 12,8 | 79,42 | 72,93 | 0,173 | 0,094 | 0,67 |
| **92** | 1530,13 | 2166,94 | 1,89 | 14,82 | 45,5 | 22,7 | -0,8 | 20,9 | 98 | 84 | 130 | 119 | 65,7 | 58,5 | 7,2 | 4,9 | 16,6 | 12,5 | 64,94 | 58,28 | 0,107 | 0,075 | 0,34 |
| **93** | 1245,93 | 1579,21 | 1,90 | 4,31 | 42,2 | 19,7 | -7,4 | 18,1 | 94 | 84 | 128 | 116 | 66,5 | 55,5 | 11,2 | 7,0 | 23,9 | 17,8 | 57,38 | 55,63 | 0,133 | 0,101 | 0,56 |
| **94** | 1197,10 | 1213,24 | 1,92 | 5,11 | 40,8 | 22,3 | -3,7 | 16,3 | 94 | 84 | 124 | 111 | 68,4 | 65,4 | 6,7 | 5,0 | 23,1 | 17,6 | 65,80 | 66,23 | 0,153 | 0,117 | 0,75 |
| **95** | 922,81 | 1188,09 | 2,17 | 4,35 | 41,8 | 22,0 | -4,5 | 17,6 | 88 | 83 | 121 | 108 | 48,7 | 51,9 | 8,6 | 6,6 | 23,2 | 17,2 | 54,40 | 45,56 | 0,123 | 0,080 | 0,41 |
| **96** | 1171,50 | 1506,88 | 2,22 | 3,23 | 45,9 | 22,1 | -6,1 | 18,7 | 97 | 84 | 127 | 114 | 54,3 | 51,9 | 8,3 | 5,9 | 18,4 | 14,8 | 78,83 | 72,41 | 0,145 | 0,094 | 0,57 |
| **97** | 1040,50 | 1304,20 | 2,22 | 7,05 | 47,9 | 19,2 | -8,0 | 24,6 | 91 | 84 | 125 | 110 | 71,7 | 64,6 | 11,2 | 8,2 | 26,4 | 18,5 | 62,34 | 53,03 | 0,160 | 0,099 | 0,66 |
| **98** | 769,52 | 1145,36 | 2,06 | 2,71 | 45,0 | 20,0 | -9,0 | 19,4 | 92 | 83 | 124 | 111 | 54,9 | 53,6 | 8,9 | 7,4 | 23,3 | 16,5 | 73,76 | 67,15 | 0,171 | 0,113 | 0,81 |
| **99** | 1317,23 | 1844,32 | 2,07 | 11,87 | 37,7 | 20,0 | 0,5 | 22,4 | 97 | 85 | 123 | 115 | 56,3 | 56,0 | 8,0 | 5,6 | 17,8 | 12,6 | 67,16 | 70,26 | 0,117 | 0,090 | 0,44 |
| **100** | 1447,73 | 2100,53 | 1,50 | 24,63 | 44,7 | 25,9 | -6,1 | 15,2 | 96 | 88 | 129 | 119 | 59,7 | 52,6 | 6,8 | 3,8 | 16,3 | 10,0 | 71,73 | 73,27 | 0,112 | 0,073 | 0,34 |

51* genotype 51 not available in 2020
