# Supplementary figures and images for: Variability in drought stress response in a panel of 100 faba bean genotypes
Source: Front Plant Sci. 2023 Aug 30;14:1236147. doi: 10.3389/fpls.2023.1236147 (PMC10499557; doi:10.3389/fpls.2023.1236147)

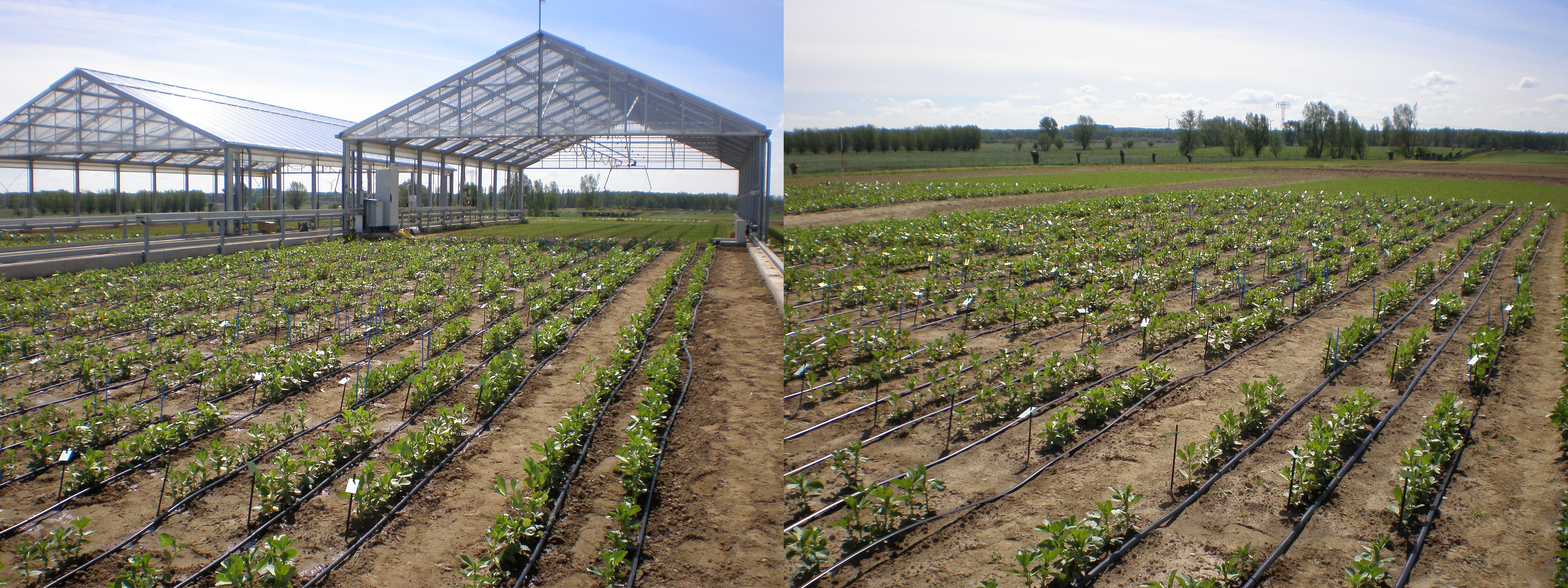

Supplement: Supplementary file 4 [file Image_1.jpeg]
